# Modular Oxidation of Cytosine Modifications and Their Application in Direct and Quantitative Sequencing of 5-Hydroxymethylcytosine

**DOI:** 10.1021/jacs.3c01663

**Published:** 2023-03-24

**Authors:** Haiqi Xu, Jinfeng Chen, Jingfei Cheng, Linzhen Kong, Xiufei Chen, Masato Inoue, Yibin Liu, Skirmantas Kriaucionis, Meiping Zhao, Chun-Xiao Song

**Affiliations:** † Ludwig Institute for Cancer Research, Nuffield Department of Medicine, University of Oxford, Oxford OX3 7FZ, U.K.; ‡ Target Discovery Institute, Nuffield Department of Medicine, University of Oxford, Oxford OX3 7FZ, U.K.; § Beijing National Laboratory for Molecular Sciences and MOE Key Laboratory of Bioorganic Chemistry and Molecular Engineering, College of Chemistry and Molecular Engineering, Peking University, Beijing 100871, China; ∥ College of Chemistry and Molecular Sciences, Wuhan University, Wuhan 430072, China; ⊥ Taikang Center for Life and Medical Sciences, Wuhan University, Wuhan 430072, China

## Abstract

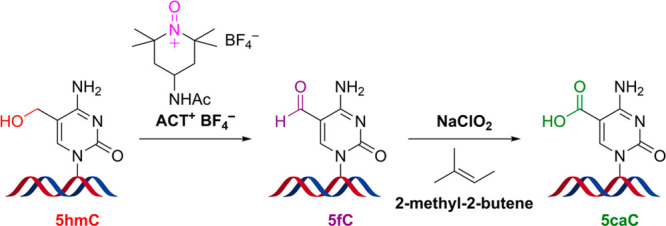

Selective, efficient, and controllable oxidation of cytosine modifications is valuable for epigenetic analyses, yet only limited progress has been made. Here, we present two modular chemical oxidation reactions: conversion of 5-hydroxymethylcytosine (5hmC) into 5-formylcytosine (5fC) using 4-acetamido-2,2,6,6-tetramethylpiperidine-1-oxoammonium tetrafluoroborate (ACT^+^BF_4_^–^) and further transformation of 5fC into 5-carboxycytosine (5caC) through Pinnick oxidation. Both reactions are mild and efficient on double-stranded DNA. We integrated these two oxidations with borane reduction to develop chemical-assisted pyridine borane sequencing plus (CAPS+), for direct and quantitative mapping of 5hmC. Compared with CAPS, CAPS+ improved the conversion rate and false-positive rate. We applied CAPS+ to mouse embryonic stem cells, human normal brain, and glioblastoma DNA samples and demonstrated its superior sensitivity in analyzing the hydroxymethylome.

5-Methylcytosine (5mC) and 5-hydroxymethylcytosine (5hmC) are the most important DNA modifications in the mammalian genome and play important roles in many biological processes.^1−3^ 5mC can be oxidized into 5hmC, 5-formylcytosine (5fC), and 5-carboxycytosine (5caC) sequentially by ten–eleven translocation (TET) enzymes^4−7^ (Figure 1a). TET proteins have been extensively used in epigenetic sequencing of 5mC and 5hmC, such as TET-assisted bisulfite sequencing (TAB-seq),^8^ Enzymatic methyl-seq (EM-seq),^9^ and TET-assisted pyridine borane sequencing (TAPS).^10^ However, TET enzymes have several limitations, including substrate preference for 5mC,^11,12^ sequence preference for methylated CpG sites,^11,13^ and relatively high cost to produce.^8−10^ More importantly, TET oxidation of 5mC cannot be easily controlled to stop at 5hmC or 5fC.^6,7,10^ Consequently, enzymatic oxidation by TET proteins may not fully meet the requirements for highly flexible transformation and manipulation of cytosine modifications *in vitro*.

**Figure 1:**
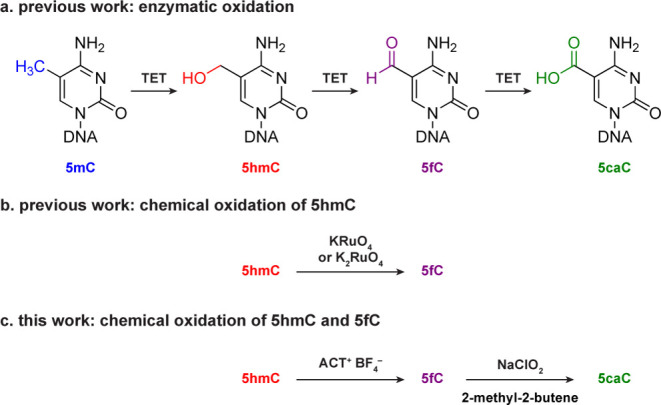
Comparison of different approaches for oxidation of cytosine modifications. (a) Enzymatic oxidation of 5mC, 5hmC, and 5fC by TET proteins. (b) Ruthenium-based oxidation of 5hmC to 5fC. (c) ACT^+^BF_4_^–^ oxidation of 5hmC to 5fC and Pinnick oxidation of 5fC to 5caC.

An ideal tool for transformation of cytosine modifications should be selective, efficient, double-stranded DNA-friendly, and easy to access. Therefore, selective chemical oxidation of cytosine modifications is highly desirable as an alternative to enzymatic oxidation through TET enzymes. Currently, the most popular chemistry is the ruthenium-based oxidation of 5hmC to 5fC, including potassium perruthenate (KRuO_4_) and potassium ruthenate (K_2_RuO_4_), which was demonstrated in oxidative bisulfite sequencing (oxBS)^14^ and chemical-assisted C-to-T conversion of 5hmC sequencing (hmC-CATCH),^15^ respectively (Figure 1b). However, the ruthenium-based oxidation only works on single-stranded DNA, thereby demanding an alkaline pre-denaturing treatment, which makes DNA prone to damage.^14^ Unlike 5hmC, effective chemical oxidation of 5mC and 5fC has not been established,^16,17^ possibly due to the intrinsically inert nature of these two modifications. Therefore, selective chemistry that can provide modular solutions to oxidation of cytosine modifications is still in high demand. Here, we present new chemical tools for selective oxidation of 5hmC to 5fC and further oxidation of 5fC to 5caC (Figure 1c). By combining these two reactions with borane reduction, we developed chemical-assisted pyridine borane sequencing plus (CAPS+), as a new version of CAPS,^18^ for highly sensitive direct and quantitative sequencing of 5hmC.

The weaknesses of ruthenium-based oxidants can be attributed to the conflict between negatively charged active species (perruthenate RuO_4_^–^ and ruthenate RuO_4_^2–^) and double-stranded DNA. To overcome these limitations, we reasoned that TEMPO-derived oxoammonium salt (TEMPO = 2,2,6,6-tetramethylpiperidine-1-oxyl) could be a better choice for selective 5hmC oxidation on double-stranded DNA under mild conditions, since this method not only uses a positively charged oxoammonium cation as the oxidative species but has also been extensively applied as a stoichiometric oxidation of alcohols in organic synthesis, displaying great efficiency and selectivity on primary allylic and benzylic alcohols.^19−23^ More importantly, in contrast to other TEMPO-catalyzed alcohol oxidations (for example, copper/TEMPO-catalyzed aerobic oxidation of alcohols^24^), this approach is free of terminal oxidants or transition metal catalysts, thus making it ideal to oxidize 5hmC specifically without causing damage to DNA.

To test the feasibility of TEMPO-derived oxoammonium salt oxidation of 5hmC, we chose ACT^+^BF_4_^–^ (also known as Bobbitt’s salt) as the oxidant, since it is stable, water-soluble, commercially available, inexpensive, and environmentally benign^22,23^ (Figure 2a). We first performed the oxidation on an 11mer oligonucleotide that contains one 5hmC site. After ACT^+^BF_4_^–^ oxidation, we found that the starting material was transformed into 5fC efficiently, which was confirmed by a 2 Da reduction in mass, detected by electrospray ionization–mass spectrometry (ESI-MS) (Figure 2b and Figure S1). In comparison, an unmodified or 5mC-labeled oligonucleotide was not oxidized under the same condition (Figure S1). Next, we applied the ACT^+^BF_4_^–^ oxidation to 79 bp double-stranded 5hmC-containing DNA and used ultra-high-performance liquid chromatography–tandem mass spectrometry (UHPLC-MS/MS) to monitor the conversion rate of 5hmC after oxidation. A 99.2% conversion was achieved under the nondenaturing condition (Figure 2c and Figure S2). We hypothesized that the mechanism of ACT^+^BF_4_^–^ oxidation involved a direct intermolecular hydride transfer step similar to previous predictions^25,26^ (Figure S3a).

**Figure 2:**
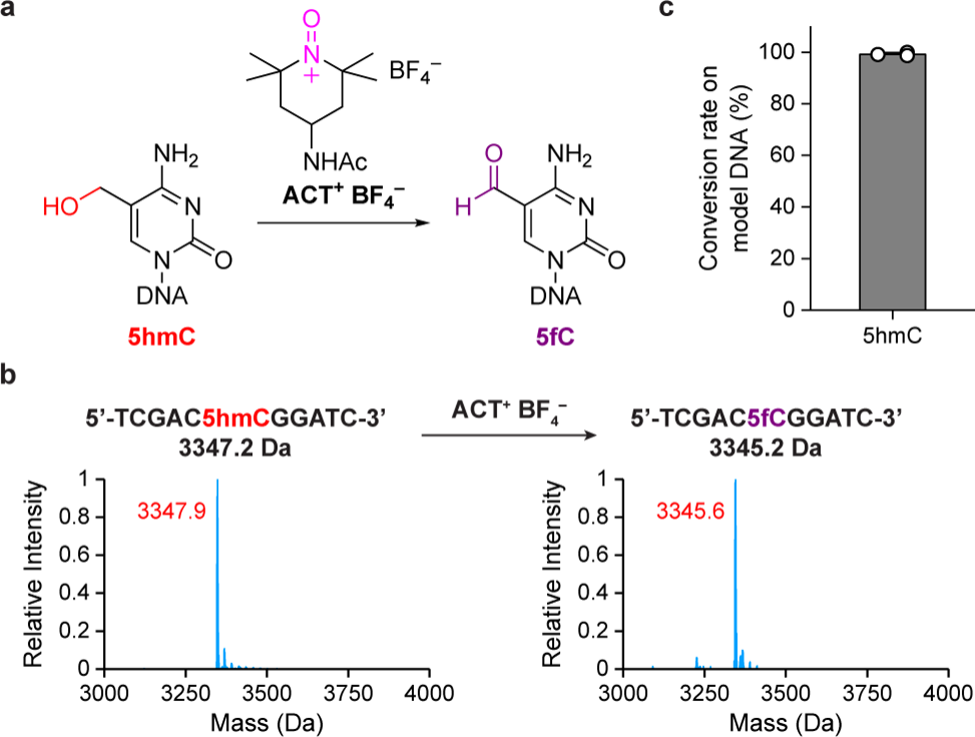
ACT^+^BF_4_^–^ oxidation of 5hmC to 5fC. (a) Schematic overview of ACT^+^BF_4_^–^ oxidation. (b) ESI-MS characterization of an 11mer 5hmC-containing oligonucleotide treated with ACT^+^BF_4_^–^. Calculated mass is shown in black. Deconvoluted mass is shown in red. (c) Conversion rate of ACT^+^BF_4_^–^ oxidation of 5hmC on 79 bp model DNA quantified by UHPLC-MS/MS. Data are shown as mean ± s.d. of three independent experiments (*n* = 3).

After we achieved the ACT^+^BF_4_^–^ oxidation of 5hmC to 5fC, we aimed to develop a novel tool to convert 5fC into 5caC. Since 5fC has a low equilibrium constant for formation of gem-diol species^27^ (Figure S3b), we focused on selective oxidation of aldehyde with special mechanisms that can circumvent the aldehyde hydration process. We envisioned that the Pinnick oxidation using sodium chlorite (NaClO_2_) would be most suitable for this purpose, since it can work on sterically hindered aldehydes and is known to tolerate a wide range of functional groups, including alcohols, electron-deficient aromatic amines, and heterocycles^28−31^ (Figure 3a and Figure S4).

**Figure 3:**
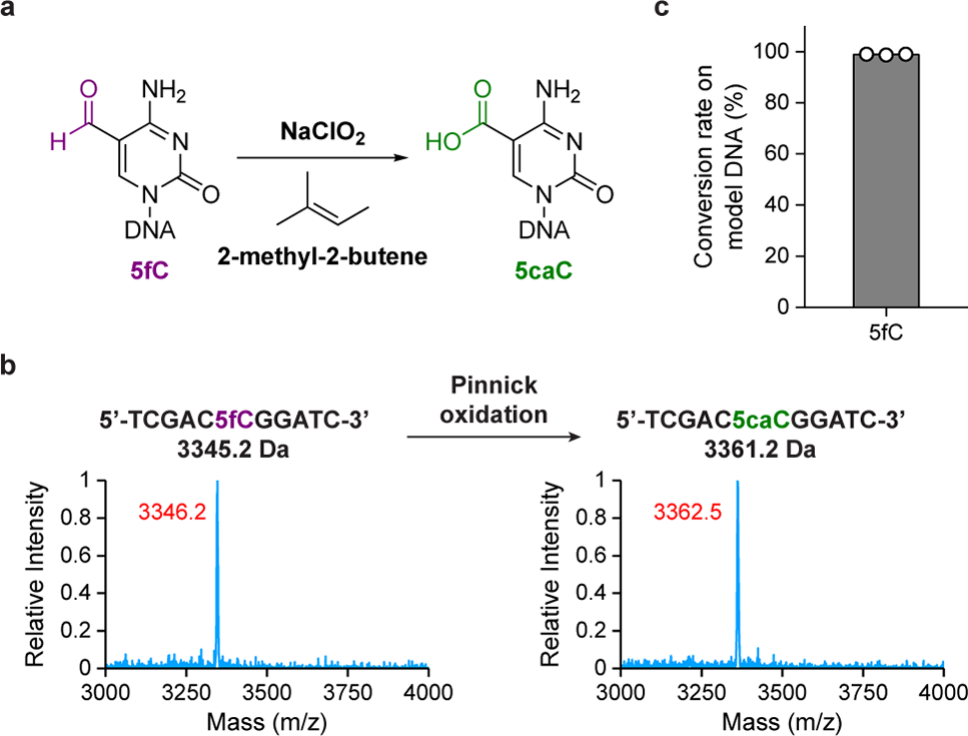
Pinnick oxidation of 5fC to 5caC. (a) Schematic overview of Pinnick oxidation using NaClO_2_ as the oxidant and 2-methyl-2-butene as the scavenger. (b) MALDI characterization of Pinnick oxidation of 11mer 5fC-containing oligonucleotide. Calculated mass is shown in black. Observed mass is shown in red. (c) Conversion rate of Pinnick oxidation of 5fC on 99 bp model DNA quantified by UHPLC-MS/MS. Data are shown as mean ± s.d. of three independent experiments (*n* = 3).

To test the Pinnick oxidation, we started with an 11mer oligonucleotide that contains one 5fC modification and monitored the reaction by matrix-assisted laser desorption/ionization mass spectrometry (MALDI). We chose 2-methyl-2-butene as the scavenger (Figure S4), and after Pinnick oxidation, we found that 5fC was completely converted into 5caC, which was confirmed by a 16 Da increase in mass (Figure 3b). In comparison, unmodified cytosine, 5mC-labeled, or 5hmC-labeled oligonucleotides were not oxidized (Figure S5). Next, we screened the reaction conditions of Pinnick oxidation by UHPLC-MS/MS (Figures S6 and S7). Finally, nearly quantitative conversion was observed on 99 bp 5fC-contaning double-stranded DNA under the optimized reaction condition (Figure 3c). To the best of our knowledge, this is the first successful selective chemical oxidation of 5fC to 5caC on DNA.

In our recent research, we found that borane complexes could readily reduce 5fC and 5caC into dihydrouracil (DHU), which would be read as thymine (T) in PCR amplification.^10,18^ Previously, by combining K_2_RuO_4_ oxidation and borane reduction, we have developed CAPS for 5hmC sequencing^18^ (Figure 4a). However, it has a relatively low conversion rate and a high false-positive rate, possibly due to the following reasons. First, the denaturing ruthenate oxidation may not be compatible with nondenaturing borane reduction. Second, 5caC could be a better substrate for borane reduction than 5fC according to the genome-wide sequencing results of CAPS and related methods (∼95% conversion rate of 5caC, 75–85% conversion rate of 5fC),^18^ although they showed similar reactivity on short model DNA.^10^ The lower conversion rate of 5fC in borane reduction is possibly due to its intrinsic 1,2-addition reactivity, which could also explain 5fC’s low conversion in bisulfite reaction compared to 5caC^14,32^ (Figure S8). Therefore, we combined nondenaturing ACT^+^BF_4_^–^ oxidation of 5hmC and Pinnick oxidation of 5fC with borane reduction to develop CAPS+ for improved base-resolution sequencing of 5hmC (Figure 4a).

**Figure 4:**
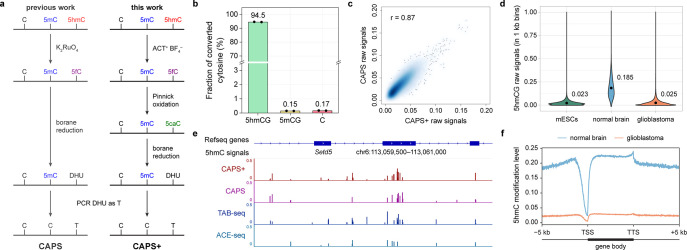
CAPS+ for quantitative and base-resolution 5hmC sequencing. (a) Comparison between CAPS and CAPS+. (b) Conversion rate for synthetic spike-in (5hmCG) and false-positive rates for CpG-methylated lambda DNA (5mCG) and 2 kb unmodified spike-in control in CAPS+. Data are shown as means of two technical replicates. (c) Correlation density plot between CAPS+ and CAPS in 10 kb bins. The color scale represents density. (d) Violin plots comparing 5hmCG raw signals in 1 kb bins between mouse embryonic stem cells (mESCs) (green), normal brain (blue), and glioblastoma (orange), with mean values listed above each plot. (e) Example of genome browser view demonstrating that CAPS+ detected consistent 5hmC sites with CAPS, TAB-seq, and ACE-seq. (f) Metagene profiles of 5hmC in normal brain (blue) and glioblastoma (orange). TSS and TTS indicate the transcription start site and the transcription termination site, respectively.

We first applied CAPS+ to fragmented mESC genomic DNA (gDNA) and compared the efficiency of two new oxidation reactions with the K_2_RuO_4_ oxidation previously used in CAPS using UHPLC-MS/MS (Figure S9). In gDNA, ACT^+^BF_4_^–^ oxidation showed higher efficiency than K_2_RuO_4_ oxidation, achieving 99.0% oxidation of 5hmC to 5fC, compared to 95.5% in K_2_RuO_4_ oxidation. Interestingly, we observed a slight increase of 8-oxoguanine (8-oxoG, one of the most common mutagenic oxidative damages^33^) in gDNA after K_2_RuO_4_ oxidation (Figure S10). In contrast, ACT^+^BF_4_^–^ and Pinnick oxidation did not lead to 8-oxoG formation, which further demonstrated their superior selectivity and mildness. As expected, CAPS+ did not cause notable DNA degradation after each step of chemical treatment (Figure S11). Based on the analysis of spike-in controls (including synthetic 5hmC spike-in, CpG-methylated lambda DNA, and 2 kb unmodified spike-in), we found that CAPS+ displayed a higher conversion rate on 5hmC (94.5%) and lower false-positive rates on 5mC (0.15%) and unmodified cytosines (0.17%) than CAPS (83.1% for 5hmC, 0.38% for 5mC, and 0.72% for unmodified cytosines)^18^ (Figure 4b). The 5hmC-to-T conversion rate of CAPS+ was comparable with the 5hmC protection rates of APOBEC-coupled epigenetic sequencing (ACE-seq) (98.5%)^34^ and TAB-seq (92.0%),^8^ while the 5mC and unmodified cytosine false-positive rates of CAPS+ were similar to or lower than the reported nonconversion rates in ACE-seq (0.5% and 0.1%, respectively)^34^ and TAB-seq (2.2% and 0.4%, respectively).^8^ Next, we examined the 5hmC raw signals from two technical replicates of CAPS+, which revealed a strong correlation between them (Pearson’s *r* = 0.85) (Figure S12). We then merged reads from two replicates and found that CAPS+ showed good correlation with CAPS (Pearson’s *r* = 0.87) (Figure 4c) and other published data sets (Pearson’s *r* = 0.64 with ACE-seq and 0.79 with TAB-seq) (Figure S13). We detected a mean 5hmCG level of 2.3% with CAPS+ (Figure 4d), compared to 2.7% detected with CAPS,^18^ which could be attributed to the low false-positive rates of CAPS+. Additionally, CAPS+ maintained high sequencing quality of CAPS and therefore exceeded ACE-seq and TAB-seq in mapping rate (91.1% for CAPS+) (Table S1) (90.7% for CAPS,^18^ 71.8% for ACE-seq,^34^ and 53.4% for TAB-seq^8^), base quality (Figure S14), and sequencing coverage uniformity (Figure S15). Comparison of results from different methods demonstrated good consistency between CAPS+ and other approaches (Figure 4e). We also analyzed the genomic distribution and enrichment profile of 5hmC sites, both of which were in agreement with previous reports^8,18,34^ (Figure S16).

To further demonstrate the utility of CAPS+ in clinical samples, we applied it to generate base-resolution maps of hydroxymethylome from human normal brain and glioblastoma gDNA. In normal brain, we detected a high 5hmC level as anticipated (mean 18.5% on CpG sites) (Figure 4d). We then compared 5hmC raw signals from CAPS+ with those from TAB-seq in adult prefrontal cortex^35^ and found a good correlation between them (Pearson’s *r* = 0.89) (Figure S17). In stark contrast to the normal brain, we observed a sharp decrease of global 5hmCG level to 2.5% in glioblastoma, which was in accordance with previous studies^36^ (Figure 4d and f).

In this study, we first developed two novel chemical reactions for selective oxidation of cytosine modifications: ACT^+^BF_4_^–^ oxidation of 5hmC into 5fC and Pinnick oxidation of 5fC into 5caC. Both reactions are highly efficient and nondestructive. In addition, they are fully compatible with double-stranded DNA and are milder than ruthenium-based oxidation with less oxidative damage. These reagents are also commercially available and easy to use without the need for special preparation and storage as for the ruthenium compound.^14,15^ To demonstrate the utility of ACT^+^BF_4_^–^ oxidation and Pinnick oxidation, we further combined them with borane reduction to establish CAPS+, an updated version of CAPS, for base-resolution sequencing of 5hmC modifications. We applied CAPS+ to various biological samples and generated base-resolution hydroxymethylome in the human normal brain and glioblastoma. We expect ACT^+^BF_4_^–^ oxidation to replace ruthenium as the new standard in 5hmC oxidation, and Pinnick oxidation, by converting 5fC to 5caC, to further improve its conversion in borane reduction and bisulfite reaction. Indeed, the flexible and modular ACT^+^BF_4_^–^ oxidation and Pinnick oxidation could be combined with other downstream chemistry, including bisulfite (as in oxBS),^14^ Friedländer synthesis (as in hmC-CATCH),^15^ and enzymatic deamination,^9,34^ as valuable tools for more accessible 5mC or 5hmC sequencing.

## Data Availability

All sequencing data are available at the GEO database (accession: GSE214006). All relevant additional data have been published with the manuscript, either as part of the main text or in the supplement. The analysis scripts are available at https://github.com/lkong888/CAPSplus.

## Supporting Information Available

Experimental methods, supporting data, additional figures, tables, and notes (PDF)
