# Supplementary material for: Modular Oxidation of Cytosine Modifications and Their Application in Direct and Quantitative Sequencing of 5-Hydroxymethylcytosine
Source: J Am Chem Soc. 2023 Mar 24;145(13):7095–100. doi: 10.1021/jacs.3c01663 (PMC10080677; doi:10.1021/jacs.3c01663)
Supplement: Supplementary file 1 — ja3c01663_si_001.pdf [file ja3c01663_si_001.pdf]

Supporting Information

Modular oxidation of cytosine modifications and their application in direct and quantitative sequencing of 5-hydroxymethylcytosine

Haiqi Xu,[†,‡,§] Jinfeng Chen,[†,‡,#] Jingfei Cheng,[†,‡,#] Linzhen Kong,[†,‡,#] Xiufei Chen,[†,‡] Masato Inoue,[†,‡] Yibin Liu,[ǁ,⊥] Skirmantas Kriaucionis,[†] Meiping Zhao,[§,*] and Chun-Xiao Song[†,‡,*]

[†]Ludwig Institute for Cancer Research, Nuffield Department of Medicine, University of Oxford, Oxford OX3 7FZ, UK

[‡]Target Discovery Institute, Nuffield Department of Medicine, University of Oxford, Oxford OX3 7FZ, UK

[§]Beijing National Laboratory for Molecular Sciences and MOE Key Laboratory of Bioorganic Chemistry and Molecular Engineering, College of Chemistry and Molecular Engineering, Peking University, Beijing 100871, China

[ǁ]College of Chemistry and Molecular Sciences, Wuhan University, Wuhan 430072, China

[⊥]Taikang Center for Life and Medical Sciences, Wuhan University, Wuhan 430072, China

[#]These authors contributed equally.

*To whom correspondence should be addressed: chunxiao.song@ludwig.ox.ac.uk; mpzhao@pku.edu.cn

Methods

Preparation of model DNA

Regular and 5mC-labelled DNA oligonucleotides were purchased from IDT or Sangon Biotech. 5hmC-labelled DNA oligonucleotides were purchased from IDT or Takara. 5fC- and 5caC-labelled DNA oligonucleotides were purchased from ATDBio. Sequences of short oligonucleotides can be found in Table S2.

The 79 bp double-stranded 5hmC-containing DNA used for UHPLC-MS/MS analysis was prepared by annealing method. The 13mer and 99 bp double-stranded 5fC-containing DNA used for UHPLC-MS/MS analysis was synthesized by primer extension method. Detailed protocols and sequences for all model DNA can be found in Supplementary Note 1.

Preparation of spike-in controls and carrier DNA for CAPS+

Detailed preparation protocols and sequences of CpG-methylated lambda DNA, 2 kb unmodified spike-in control and carrier DNA can be found in previous publications[1-2]. Carrier DNA was fragmented by Covaris M220 and size-selected to 150–250 bp with 0.9–1.2× Ampure XP beads according to the manufacturer's protocol. 144mer synthetic 5hmC spike-in control was synthesized by primer extension method, which is described in Supplementary Note 2.

Mass spectrometry analysis of short oligonucleotides

ESI-MS was performed on a Vion IMS QTof mass spectrometer (Waters). The source-dependent parameters were as follows: capillary voltage 2.5 kV, source temperature 100 °C, desolvation temperature 250 °C, cone gas flow 50 l h^{-1}, desolvation gas flow 800 l h^{-1}. All the oligonucleotides were analyzed in the negative mode. A summary of calculated and recorded masses of short oligonucleotides and reaction products was shown in Table S3.

MALDI was performed on a Voyager-DE Biospectrometry Workstation (Applied Biosystems) with 2',4',6'-trihydroxyacetophenone (THAP) as matrix. All the oligonucleotides were analyzed in the positive mode.

Quantification of 5hmC, 5fC and 5caC level on model DNA by UHPLC-MS/MS

The oxidized model DNA was digested into nucleosides by Nucleoside Digestion Mix (New England Biolabs) in 20 µl solution according to the manufacturer's protocol. The mixture was diluted to 100 µl and centrifuged at 12,000× g and 4 °C for 20 min. Finally, 2–3 µl of the supernatant was injected into UHPLC-MS/MS.

The nucleosides were separated by ultra-high performance liquid chromatography on an ACQUITY UPLC BEH C18 column (2.1×100 mm, 1.7 µm, Waters) and detected by LCMS-8050 triple quadrupole mass spectrometer (Shimadzu) in the positive ion multiple reaction monitoring (MRM) mode. The column temperature was maintained at 30 °C, and the solvent system was water containing 10 mM ammonium acetate (pH 7.0, solvent A) and methanol (solvent B) with 0.4 ml min^{-1} flow rate. The gradient was: 0–1.50 min, 0% solvent B; 1.50–4.50 min, 0–10% solvent B; 4.50–5.00 min, 10–30% solvent B; 5.00–5.01 min, 30–100% solvent B; 5.01–7.00 min, 100% solvent B; 7.00–7.01 min, 100–0% solvent B; 7.01–9.50 min, 0% solvent B. The source-dependent parameters were as follows: nebulizing gas flow 3 l min^{-1}, heating gas flow 10 l min^{-1}, interface temperature 300 °C, desolvation line temperature 250 °C, heat block temperature 400 °C, drying gas flow 10 l min^{-1}. The ions were monitored in positive mode with mass transitions of m/z

258 to 142 (hmdC+H), m/z 278 to 162 (fdC+Na) and m/z 294 to 178 (cadC+Na) (Table S4). Concentrations of nucleosides in DNA samples were deduced by fitting the signal peak areas into the standard curves.

Quantification of 5hmC, 5fC, 5caC and 8-oxoG level on gDNA by UHPLC-MS/MS

K_2RuO_4 oxidation was performed according to previous publications[3-4]. The oxidized gDNA was digested into nucleosides by Nucleoside Digestion Mix (New England Biolabs) in 20 μl solution according to the manufacturer's protocol. After filtering with Amicon Ultra-0.5 mL 3K centrifugal filters (Millipore), the digested samples were subjected to UHPLC–MS/MS analysis as described before[5]. 1290 Infinity LC Systems (Agilent) was equipped with a ZORBAX RRHD SB-C18 column (2.1 × 150 mm, 1.8 μm, Agilent) coupled with a 6495B Triple Quadrupole Mass Spectrometer (Agilent). The ions were monitored in positive mode with mass transitions of m/z 258 to 142 (hmdC+H), m/z 278 to 162 (fdC+Na), m/z 294 to 178 (cadC+Na) and m/z 284 to 168 (8-oxodG+H) (Table S5). Concentrations of nucleosides in DNA samples were deduced by fitting the signal peak areas into the standard curves.

mESCs culture and isolation of genomic DNA

E14 mESCs (gift from S. Kriaucionis) were cultured on gelatin-coated plates in DMEM (Invitrogen) supplemented with 15% FBS (Gibco), 2 mM L-glutamine (Gibco), 1% nonessential amino acids (Gibco), 1% penicillin/streptavidin (Gibco), 0.1 mM β-mercaptoethanol (Sigma-Aldrich), 1,000 units ml^{-1} leukemia inhibitory factor (Millipore), 1 μM PD0325901 (Stemgent) and 3 μM CHIR99021 (Stemgent). Cultures were maintained at 37 °C and 5% CO_2 and passaged every 2 d. For isolation of genomic DNA, cells were harvested by centrifugation for 5 min at 1,000× g and room temperature. DNA was extracted with Quick-DNA Plus kit (Zymo Research) according to the manufacturer's protocol.

Human normal brain and glioblastoma samples

The gDNA of human normal brain and glioblastoma samples were obtained from the Amsbio. Detailed clinical information for each sample is provided in Table S1.

ACT^+ BF_4^- oxidation of 5hmC to 5fC

100 ng ligated DNA was incubated in 25 μl solution containing 50 mM sodium phosphate buffer (pH 7.5) and 50 mM ACT^+ BF_4^- (TCI) at 37 °C for 4 h. The oxidized DNA was purified with 1.8× AMPure XP Beads.

5fC labelling by hydroxylamine

14 μl oxidized 11mer 5hmC-containing DNA was added to a 20 μl reaction containing 0.1 M MES buffer (pH 5.0), 10 mM *O*-ethylhydroxylamine (Sigma-Aldrich) and incubated at 37 °C for 2 h. The product was purified using Micro Bio-Spin P-6 SSC column (Bio-Rad, washed four times with water before use).

Pinnick oxidation of 5fC to 5caC

5 M 2-methyl-2-butene (Acros) was prepared by mixing the pure compound (~9.4 M) with 100% ethanol. The converted DNA was added to a 30 μl reaction containing 0.2 M sodium acetate buffer (pH 4.3), 0.16 M $NaClO_2$ (Alfa Aesar) and 1 M 2-methyl-2-butene. The reaction was then incubated at 25 °C for 16 h and double purified with Micro Bio-Spin P-6 SSC column (Bio-Rad, washed four times with water before use) and 1.8× AMPure XP Beads.

CAPS+ library construction and sequencing

mESC, human normal brain and glioblastoma gDNA was spiked with 0.5% of CpG-methylated lambda DNA and 0.25% of unmodified 2 kb spike-in control. DNA samples were fragmented by Covaris M220 and size-selected to 300–500 bp with 0.55–0.85× Ampure XP beads according to the manufacturer's protocol. The sonicated DNA was additionally spiked with 0.05% of 144mer spike-in after size-selection with Ampure XP beads. End-repair and A-tailing reaction and ligation of NEBNext Adaptor for Illumina were prepared with KAPA HyperPrep Kit according to the manufacturer's protocol. The uracil in the loop of NEBNext Adaptor was removed by adding 6 μl of USER Enzyme (New England Biolabs) to the ligation reaction and incubating at 37 °C for 45 min. The reaction was purified with 0.8× Ampure XP beads by washing twice with 80% acetonitrile/water (v/v). ACT^+ BF_4^- oxidation and Pinnick oxidation were performed as described above. Borane reduction was then performed in a 50 μl reaction using optimized condition at 37 °C and 850 r.p.m. for 6 h and purified by Zymo-IC column with Oligo Binding Buffer[4]. Converted DNA was amplified with NEBNext Multiplex Oligos for Illumina (Dual Index Primers Set 1) and KAPA HiFi HotStart Uracil+ ReadyMix PCR Kit for 4 cycles according to the manufacturer's protocols. The PCR product was purified with 0.9× Ampure XP beads and quantified with Qubit dsDNA HS Assay Kit (Thermo Fisher) according to the manufacturer's protocol. Libraries were sequenced on NextSeq 2000 (150 bp paired end) with no PhiX added.

Data pre-processing

Raw sequencing reads were processed as previously reported[4]. Briefly, fastp (v.0.12.4)[6] was used to filter bad reads, trim low-quality bases and cut adaptors. Trimmed reads were then aligned to either mm9 (for mESC samples) or hg38 (for human normal brain and glioblastoma samples) reference genome using BWA mem (v.0.7.17)[7]. PCR duplicates were removed by MarkDuplicate in Picard tools (v.2.23.0) (http://broadinstitute.github.io/picard/). Methylation calling was performed by asTair (v3.3.2)[1] using reads with MAPQ > 10. Regions prone to cause mapping artefacts were excluded from subsequent analysis (https://sites.google.com/site/anshulkundaje/projects/blacklists)[8-9]. For mESC samples, known single nucleotide variants in E14 cell lines (http://epigenetics.hugef-research.org/data.php)[10] were also excluded. For human normal brain and glioblastoma samples, CpG sites overlapping with common single nucleotide polymorphisms (SNPs) (dbSNP153)[11] and centromeres[11] were further excluded from downstream analysis.

Published data

Related published data we used were downloaded from the Gene Expression Omnibus (GEO) database: CAPS (GSE155613)[4]; TAB-seq for mESCs (GSE36173)[12]; ACE-seq (GSE116016)[13]; TAB-seq for adult prefrontal cortex (GSE46710)[14]. TAB-seq data for mESCs were reprocessed as previously described to obtain modified and unmodified CpG sites[12]. Blacklisted regions and common single nucleotide variants (SNVs) were also excluded. 5hmCG methylation calls in adult prefrontal cortex were downloaded from GSE46710 and converted from hg19 to hg38 using CrossMap.py[15].

Pairwise comparison of CAPS+

To calculate correlation between two mESC replicates, 5hmCG raw signals (T/C+T) were calculated in 10 kb genomic bins. The Pearson correlation coefficient (Pearson's *r*) was calculated by cor function in R. The results were visualized by SmoothScatter in R. Pairwise comparisons between CAPS+ and CAPS, TAB-seq as well as ACE-seq were performed similarly.

Coverage analysis for CAPS+, CAPS and ACE-seq

CpG islands were divided into 10 equal-sized bins, while the 4 kb flanking regions were binned into 20 windows. Average per strand CpG coverage was calculated as the sum of modified and unmodified cytosines. Reads from two replicates of CAPS+ were merged and subsampled to the similar coverage of ACE-seq using samtools view with the parameter (–s 0.60).

Statistical testing of high confidence 5hmC sites and genomic annotations

As previously described, a binomial test was used to estimate high confidence 5hmCG sites[4]. A p-value for each site was calculated by comparing the average false-positive rate measured by 2 kb unmodified spike-in between replicates. 5hmCG sites whose per-strand coverage was lower than 5 were filtered. 5hmCG sites with Benjamini–Hochberg (BH) adjusted p-value less than 0.01 were identified to be high confidence. For annotations of high confidence 5hmCG sites, genomic features of mouse genome were downloaded (https://github.com/gireeshkbogu/chromatin_states_chromHMM_mm9)[16]. High confidence 5hmCG sites in each regulatory genomic region were found by bedtools intersect[17] and the counts in each category were normalized by genomic coverages of corresponding category regions. To investigate the enrichment of 5hmCG sites, all detected CpG sites were sampled 10 times to generate the background distribution in different genomic elements.

5hmCG profiles in gene bodies

Annotated genes were downloaded from RefSeq database (https://www.ncbi.nlm.nih.gov/refseq/MANE/). 5hmCG scores within gene bodies, 5 kb upstream of TSS and 5 kb downstream regions of transcription termination sites (TTS) sites were calculated by computeMatrix in deepTools (v.3.3.1)[18] with parameters (--beforeStartLength 5000 --regionBodyLength 5000 --afterRegionStartLength 5000 --binSize 10). 5 kb flanking regions were binned into 10 bp windows. The gene body regions were fitted into 5 kb and divided into 500 bins. 5hmC scores was calculated with CpG sites covered by at least 5 reads per strand.

Genomic view

Methylation calls in browser extensible data (BED) format were converted to bigwig (BW) format by bedGraphToBigWig[19]. 5hmCG signals in regions of interest were visualized by Integrative Genomics Viewer (IGV)[20].

Comparison of 5hmCG signals between human normal brain and glioblastoma samples

5hmCG raw signals (T/T+C) in both normal brain and glioblastoma samples were calculated in 1 kb genomic bins.

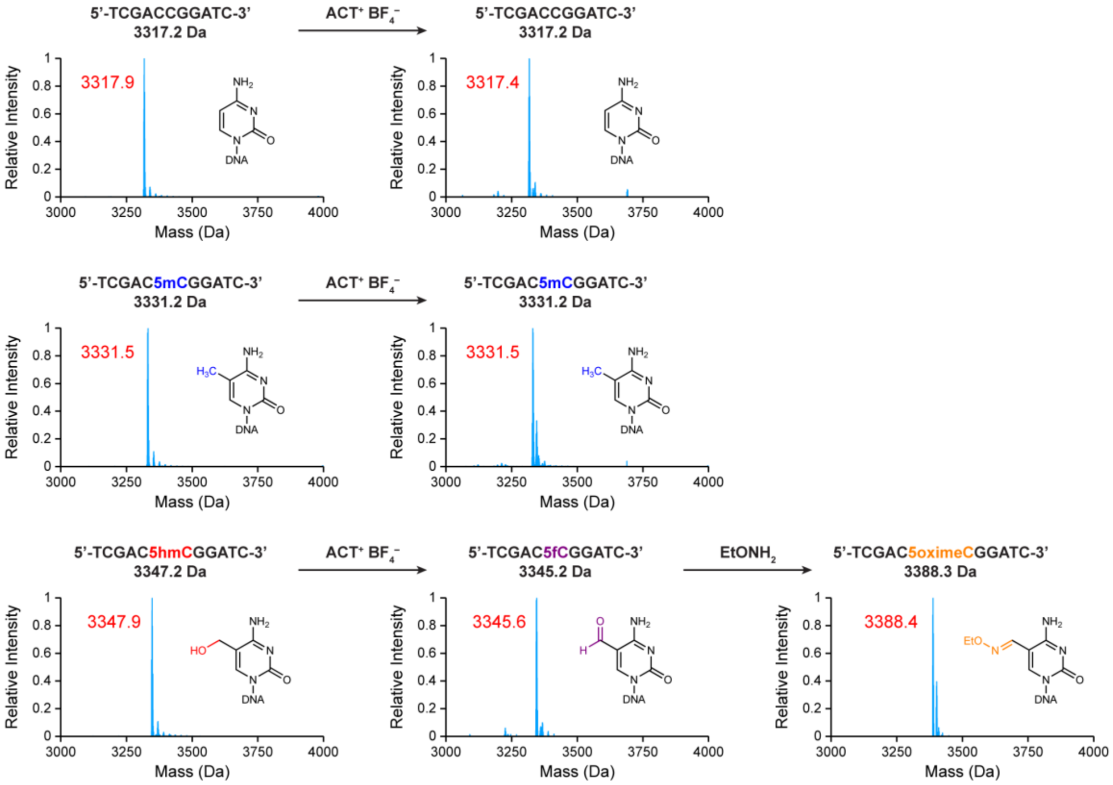

Figure S1. ESI-MS characterization of 11mer oligonucleotide treated with ACT$^+$ BF$_4^-$. To better distinguish the newly generated 5fC oligonucleotide from the 5hmC oligonucleotide, we labelled 5fC via hydroxylamine chemistry to enlarge mass differences[21]. Calculated mass is shown in black. Deconvoluted mass is shown in red. All experiments were performed once.

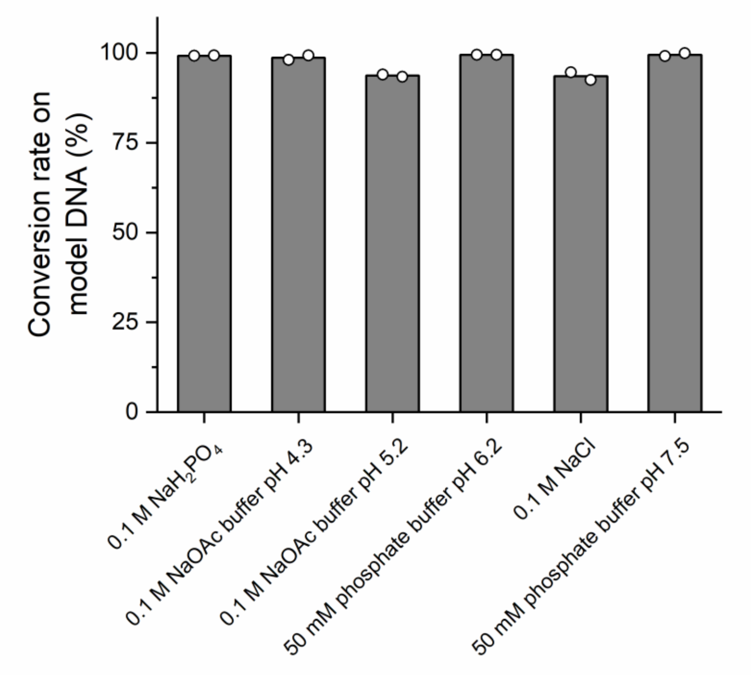

Figure S2. Buffer compatibility of ACT^+ BF_4^- oxidation of 5hmC. In addition to phosphate buffer (pH 7.5) (results also shown in Figure 2c), various buffers such as phosphate and acetate buffer with different pH were also compatible with ACT^+ BF_4^- oxidation. Reactions were conducted on 79 bp 5hmC-containing model DNA. Conversion rate was calculated by concentrations of 5hmC and 5fC. Conversion rate = $Concentration_{5fC}$ / ($Concentration_{5hmC}$ + $Concentration_{5fC}$). Data are presented as means of two independent replicates.

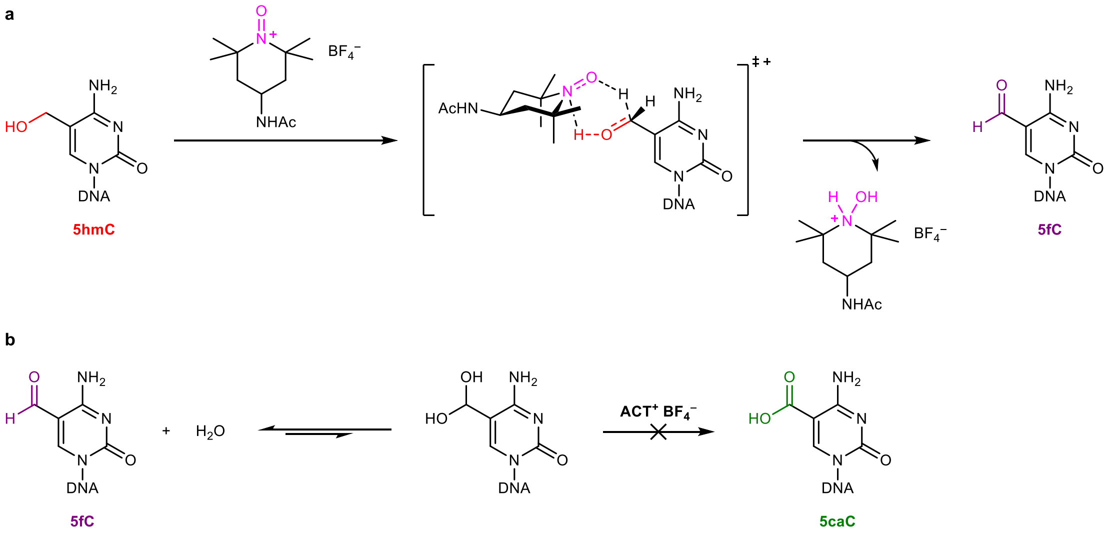

Figure S3. Mechanistic overview of ACT$^+$ BF_4^- oxidation of 5hmC. The oxidative species is ACT$^+$, a positively charged ion, which could access double-stranded DNA easily and overcome the fundamental limitations of negatively charged ruthenium-based oxidants: it is relatively difficult for RuO_4^- or RuO_4^{2-} to approach the 5hmC sites without denaturation; however, denatured DNA is more prone to damage when complete conversion of 5hmC is required, thus making it hard to find a balance between high reaction efficiency and high DNA recovery. Although some transition metal cations (such as Cu^{2+}, Ag^+ and Fe^{3+}) also bear one or more positive charges and can be involved in alcohol oxidation as a catalyst or terminal oxidant, these compounds may result in significant DNA damage due to their potential Lewis acid or redox activity[22-23]. In comparison, ACT$^+$ BF_4^- could work in a non-catalyzed and stoichiometric manner and thus could offer milder conditions for DNA oxidation. (a) Proposed mechanisms of ACT$^+$ BF_4^- oxidation of 5hmC into 5fC. A direct intermolecular hydride transfer step is involved. After oxidation, the ACT$^+$ ion would be transformed into the corresponding hydroxylammonium ion, so the pH of reaction mixture would not change significantly. (b) Schematic explanation of the resistance of 5fC towards ACT$^+$ BF_4^- oxidation. Although ACT$^+$ BF_4^- has the potential to oxidize a hydroxyl group directly to carboxylic acid[24-25], we did not observe the formation of 5caC, possibly due to the rather low hydration equilibrium constant of 5fC, particularly in a neutral aqueous solution at ambient temperature.

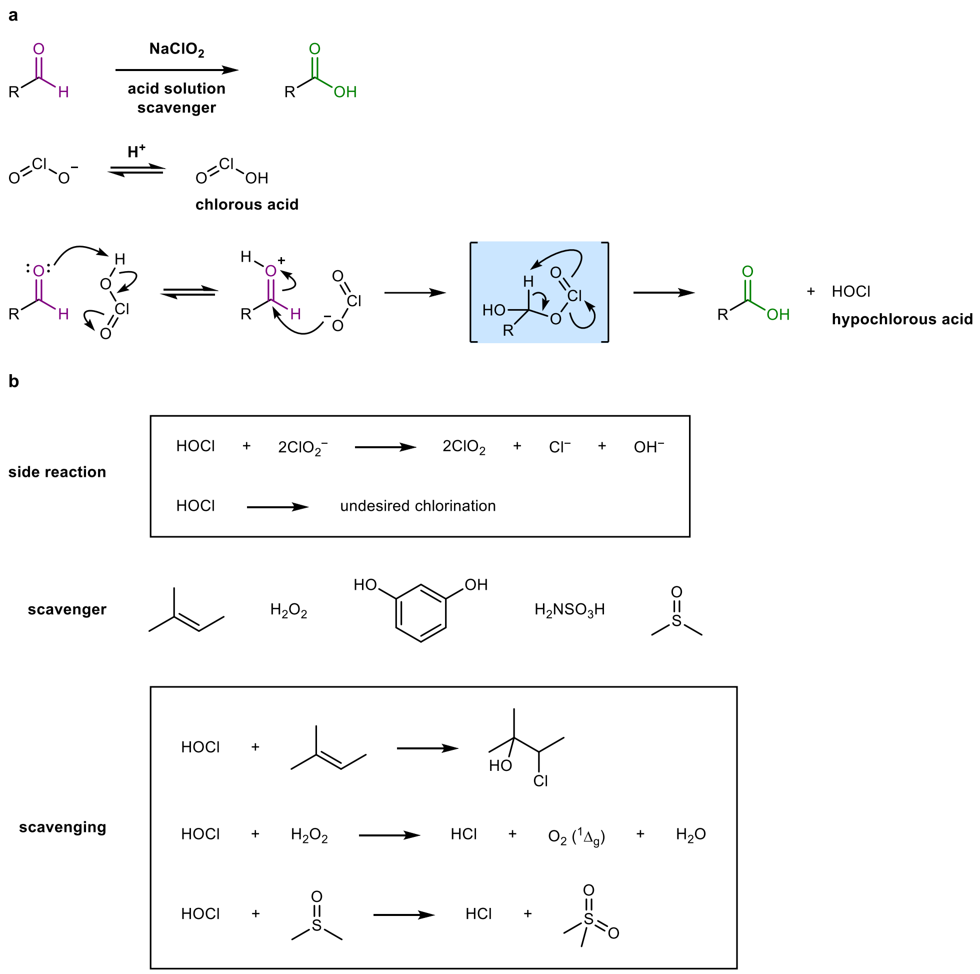

Figure S4. Mechanistic overview of Pinnick oxidation of 5fC. (a) Proposed mechanisms of Pinnick oxidation. This mechanisms suggest that chlorous acid ($HClO_2$), which is formed *in situ* by protonation of chlorite anion (ClO_2^-), would serve as the actual oxidant and react with the aldehyde group through a five-membered ring fragmentation process to generate the corresponding carboxylic acid and hypochlorous acid (HOCl) as a by-product[26]. Instead of geminal diols, a critical intermediate (shown in blue) is believed to play an important role in Pinnick oxidation. (b) Common scavengers in Pinnick oxidation and their scavenging reactions. Since HOCl is highly reactive and oxidative, it would consume ClO_2^- and lead to undesired chlorination or degradation of substrates[27]. To eliminate the negative impact of HOCl, scavengers which are more reactive towards HOCl than the substrates would be added into the reaction system in large excess. However, only 2-methyl-2-butene and dimethyl sulfoxide (DMSO) have been tested, because both these compounds and their scavenging products would not interfere with Pinnick oxidation or induce damage to DNA. Sulfamic acid will influence the pH of reaction mixture. Phenol-type scavengers (such as resorcinol) are not stable enough to air. Hydrogen peroxide (H_2O_2) could cause oxidative damage to DNA, and it would also react with HOCl to generate highly reactive singlet molecular oxygen (O_2 $^1\Delta_g$).

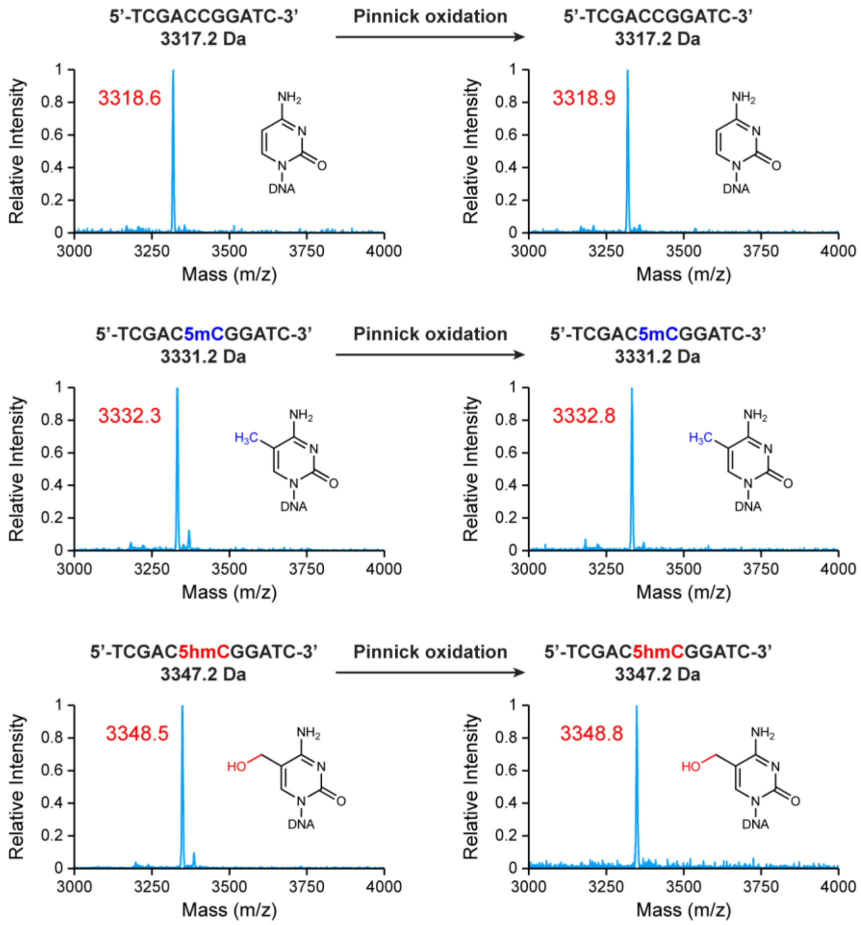

Figure S5. MALDI characterization of Pinnick oxidation of 11mer oligonucleotide. Calculated mass is shown in black. Observed mass is shown in red. All experiments were performed once.

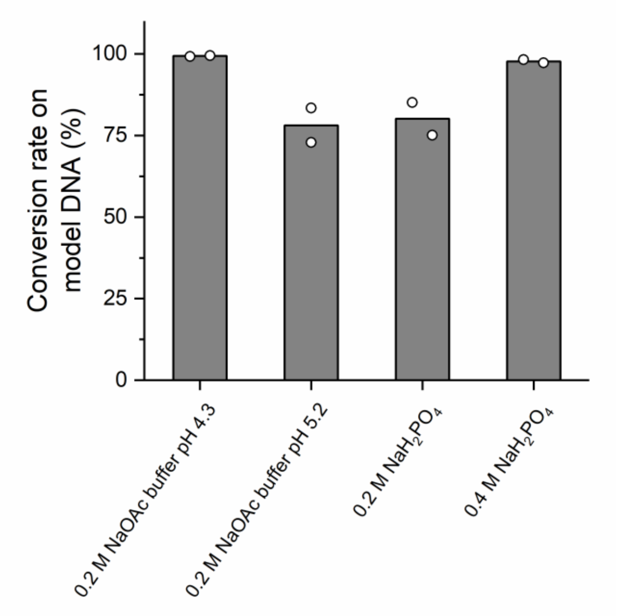

Figure S6. Optimization of reaction solution of Pinnick oxidation of 5fC. As predicted, Pinnick oxidation of 5fC only proceeded efficiently in an acidic solution with pH below 5.0. Acetate buffer was better than the same concentration of monosodium phosphate (NaH_2PO_4) solution when working as the proton source. Reactions were conducted on 13mer 5fC-containing model DNA. Conversion rate was calculated by concentrations of 5fC and 5caC. Conversion rate = $Concentration_{5caC}$ / ($Concentration_{5fC}$ + $Concentration_{5caC}$). Data are presented as means of two independent replicates.

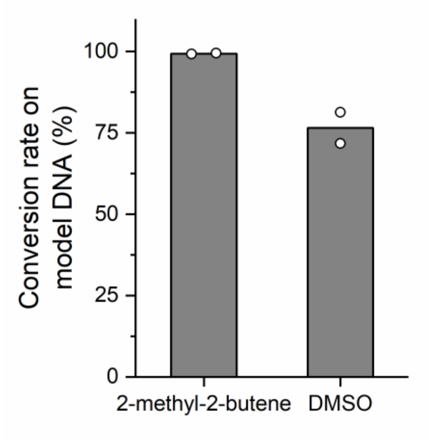

Figure S7. Comparison of scavengers in Pinnick oxidation of 5fC. DMSO can work as a non-canonical scavenger in Pinnick oxidation of 5fC[28], but the UHPLC-MS/MS results suggested that DMSO could not remove the side product HOCl efficiently, as the conversion rate of 5fC was lower. Reactions were conducted on 13mer 5fC-containing model DNA. Conversion rate was calculated by concentrations of 5fC and 5caC. Conversion rate = $\text{Concentration}_{5caC}$ / ($\text{Concentration}_{5fC}$ + $\text{Concentration}_{5caC}$). Data are presented as means of two independent replicates.

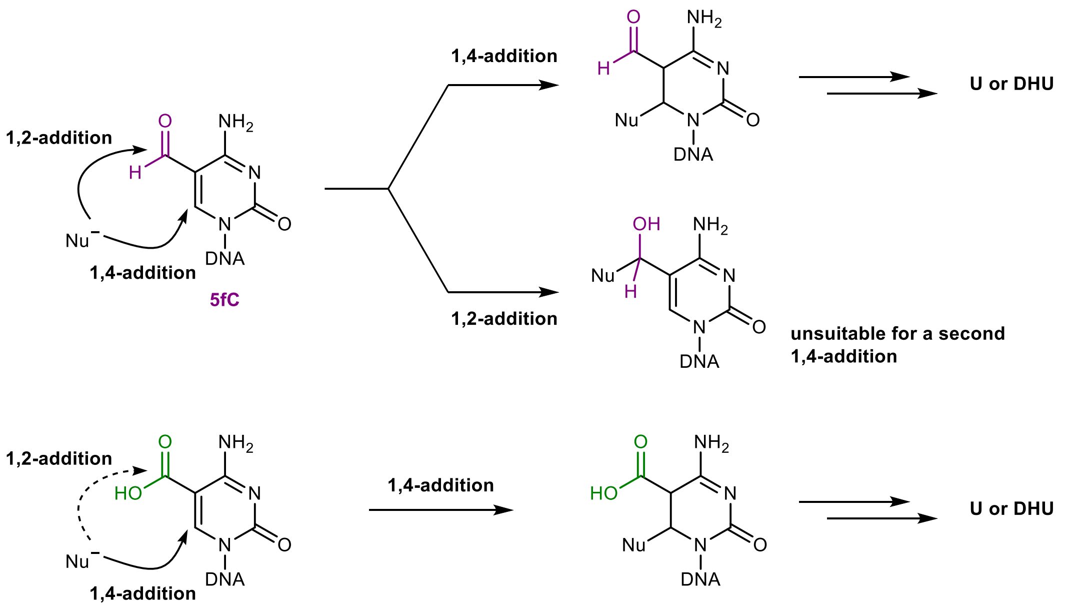

Figure S8. Schematic overview of reactivity of 5fC and 5caC towards soft nucleophile. Since both borane reduction and bisulfite-mediated deamination start with a critical conjugate addition (1,4-addition) of the C^5-C^6 double bond of the pyrimidine ring, potential 1,2-addition reactivity of 5fC may result in incomplete conversion: the 1,2-adduct is much less electron-deficient than 5fC and therefore unsuitable for a second 1,4-addition. Compared to the formyl group in 5fC, the carboxylic acid group in 5caC is much more inert and less likely to undergo 1,2-addition. As a result, 5fC could serve as a worse substrate than 5caC in borane reduction and bisulfite sequencing.

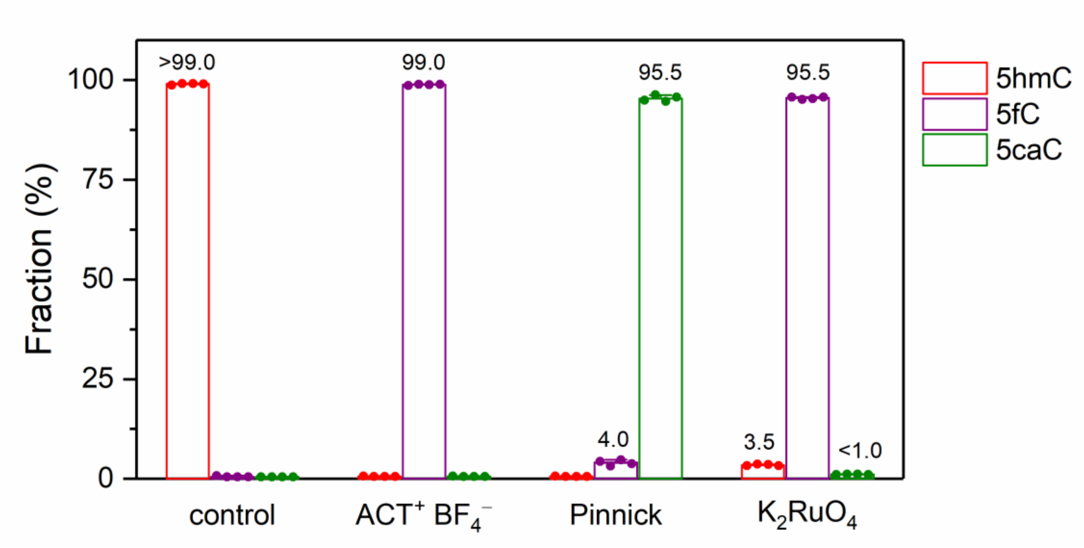

Figure S9. Comparisons of efficiency of ACT^+ BF_4^- oxidation, Pinnick oxidation (both used in CAPS+) and ruthenium-based oxidation (used in CAPS) on 300–500 bp fragmented mESC gDNA. In addition to complete transformation of 5hmC to 5fC by ACT^+ BF_4^- oxidation, combining it with Pinnick oxidation further achieved 95.5% oxidation of 5hmC to 5caC, which was comparable to the efficiency of TET oxidation[1]. Data are shown as mean ± s.d. of four independent experiments (n = 4).

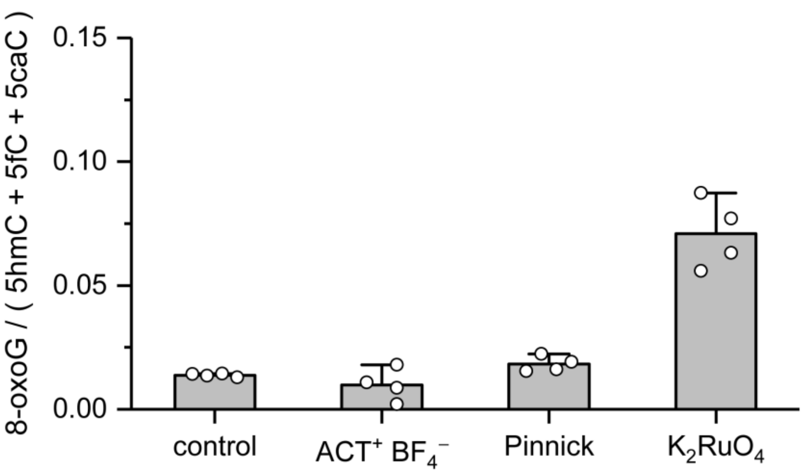

Figure S10. Comparisons of 8-oxoG formation after $ACT^+ BF_4^-$ oxidation, Pinnick oxidation (both used in CAPS+) and ruthenium-based oxidation (used in CAPS). 8-oxoG level is normalized to the sum of oxidized cytosine modification level (5hmC + 5fC + 5caC). Data are shown as mean ± s.d. of four independent experiments (n = 4).

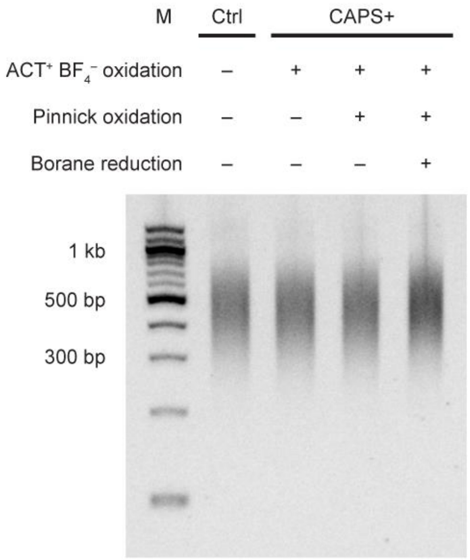

Figure S11. No detectable degradation of ligated DNA was observed on 2% agarose gel after CAPS+. Experiment was performed once.

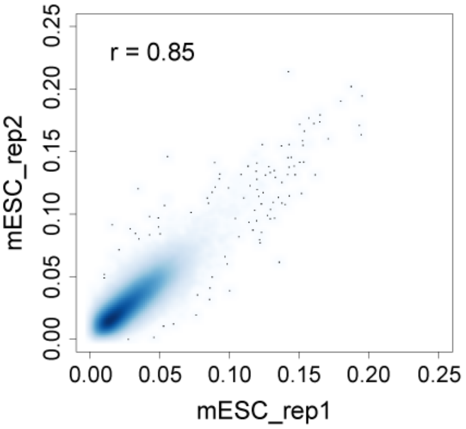

Figure S12. Correlation density plot between two technical replicates of CAPS+ in 10 kb bins. The color scale represents density.

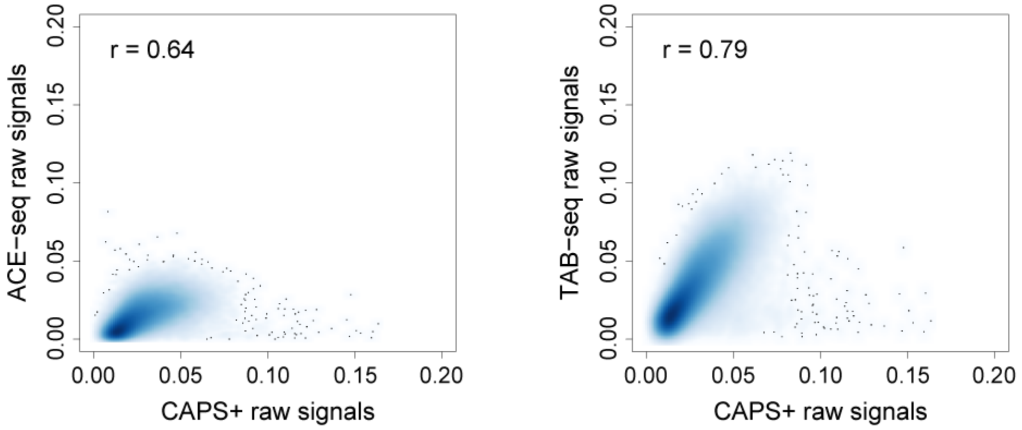

Figure S13. Correlation density plot between CAPS+, ACE-seq, and TAB-seq in 10 kb bins. The color scale represents density.

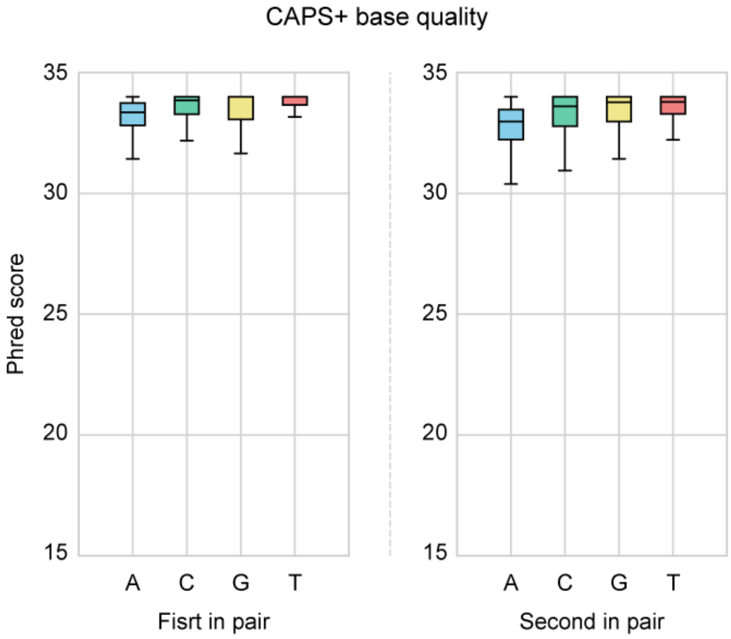

Figure S14. Sequencing base quality of CAPS+. CAPS+ showed high sequencing quality scores per base for the first and second reads in all sequenced read pairs. Reads from two replicates of CAPS+ were merged. Nucleotide is denoted by color. Boxplots visualize all sequencing reads showing medians, upper and lower fourth quantiles and non-outlier extreme values (n = 10 million random sequencing reads).

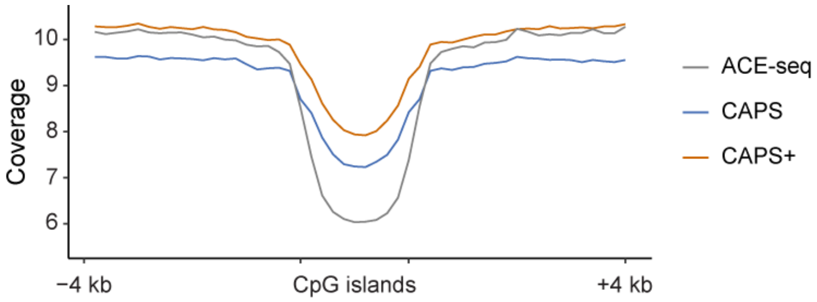

Figure S15. Average sequencing coverage of CAPS+, CAPS and ACE-seq at all CpG islands (CGI) and 4 kb flanking regions. CAPS+ maintained good sequencing coverage of CAPS and surpassed ACE-seq. Reads from two replicates of CAPS+ were merged and subsampled to the similar coverage of ACE-seq.

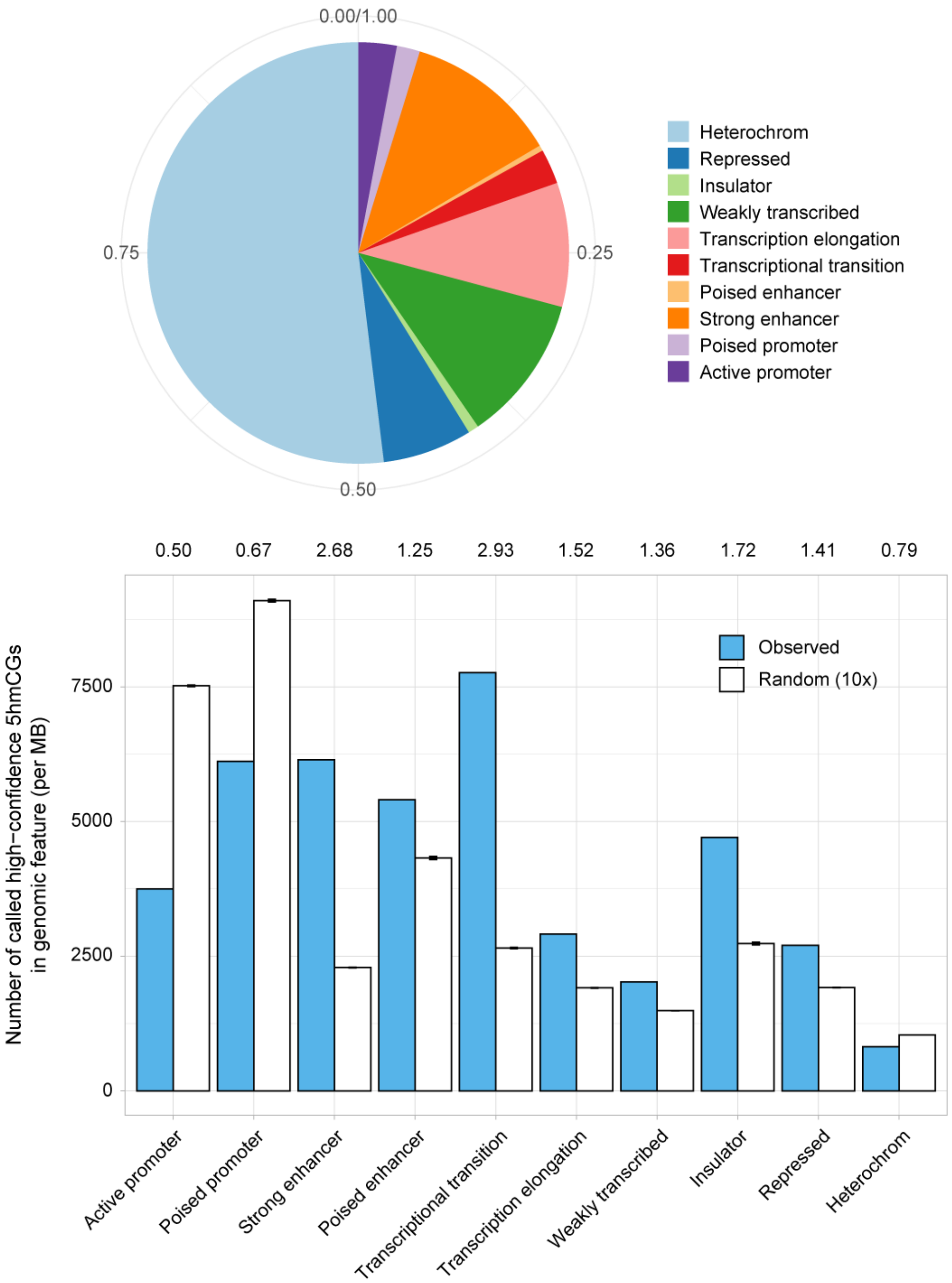

Figure S16. Pie chart shows the overlap of called 5hmCGs with putative genomic regulatory elements and the relative enrichment of 5hmCGs (blue) and random sites (white) at genomic regulatory elements in mESC. 'Random' consists of ten random samplings. The mean is shown as the bar height and the error bars denote standard deviation (n = 10 random sampling events). The ratios between observed and random are shown at the top.

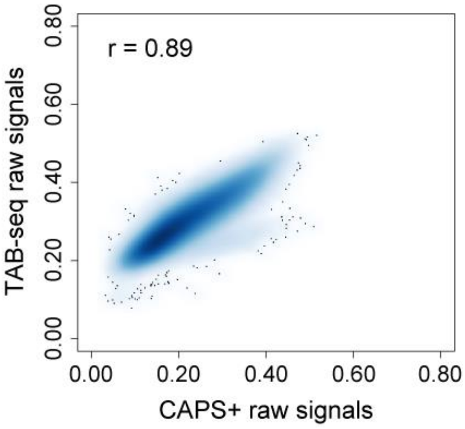

Figure S17. Correlation density plot between CAPS+ signals in human normal brain and TAB-seq signals in adult prefrontal cortex in 10 kb bins. The color scale represents density.

Table S1. Sequencing metrics and sample information.

Sample	Total sequenced reads	Mapped reads (MAPQ > 10)	Mapping rate (%)	Duplication rate (%)	5hmCG conversion rate (%)	5mCG false-positive rate (%)	C false-positive rate (%)
mESC_rep1	250662830	226330701	91.1%	39.2%	94.4%	0.14%	0.16%
mESC_rep2	250677114	226426441	91.1%	37.8%	94.6%	0.15%	0.17%
human normal brain	397161527	381982638	96.9%	37.2%	94.2%	0.15%	0.20%
glioblastoma	313795080	294639533	94.6%	36.7%	91.8%	0.12%	0.15%

Sample	Species	Cell-type (s)	Donor sex	Donor Age	Donor race	Donor pathology
mESC_rep1	mouse	embryonic stem cells				
mESC_rep2	mouse	embryonic stem cells				
human normal brain	human	tissue	male	77	Caucasian	normal
glioblastoma	human	tissue	female	60	Asian	glioblastoma

Table S2. Short DNA oligonucleotide sequences in this work.

Name	Sequence (5' to 3')	Source
for ESI-MS		
11 mer C-ODN	TCGAC**C**GGATC	Sangon Biotech
11 mer 5mC-ODN	TCGAC**5mC**GGATC	Sangon Biotech
11 mer 5hmC-ODN	TCGAC**5hmC**GGATC	Takara
for MALDI		
11 mer C-ODN	TCGAC**C**GGATC	IDT
11 mer 5mC-ODN	TCGAC**5mC**GGATC	IDT
11 mer 5hmC-ODN	TCGAC**5hmC**GGATC	IDT
11 mer 5fC-ODN	TCGAC**5fC**GGATC	ATDBio

Table S3. Calculated and recorded masses of short oligonucleotides and reaction products.

Name	Calculated mass (Da)	Calculated ion m/z	ESI-MS found
11 mer C-ODN	3317.2	$[M–2H]^{2–}$ = 1657.6<br>$[M–3H]^{3–}$ = 1104.7<br>$[M–4H]^{4–}$ = 828.3<br>$[M–5H]^{5–}$ = 662.4	1657.31<br>1104.54<br>828.15<br>662.32
11 mer 5mC-ODN	3331.2	$[M–2H]^{2–}$ = 1664.6<br>$[M–3H]^{3–}$ = 1109.4<br>$[M–4H]^{4–}$ = 831.8<br>$[M–5H]^{5–}$ = 665.2	1664.46<br>1109.31<br>831.73<br>665.18
11 mer 5hmC-ODN	3347.2	$[M–2H]^{2–}$ = 1672.6<br>$[M–3H]^{3–}$ = 1114.7<br>$[M–4H]^{4–}$ = 835.8<br>$[M–5H]^{5–}$ = 668.4	1672.30<br>1114.54<br>835.65<br>668.32
11 mer 5fC-ODN	3345.2	$[M–2H]^{2–}$ = 1671.6<br>$[M–3H]^{3–}$ = 1114.1<br>$[M–4H]^{4–}$ = 835.3<br>$[M–5H]^{5–}$ = 668.0	1671.30<br>1113.86<br>835.14<br>667.92
11 mer 5oximeC-ODN	3388.3	$[M–2H]^{2–}$ = 1693.2<br>$[M–3H]^{3–}$ = 1128.4<br>$[M–4H]^{4–}$ = 846.1<br>$[M–5H]^{5–}$ = 676.6	1692.81<br>1128.20<br>845.90<br>676.52

Table S4. Compound-dependent UHPLC-MS/MS parameters used for nucleosides quantification on model DNA. All the nucleosides were analyzed in the positive mode.

Compound	Precursor Ion (m/z)	Product Ion (m/z)	RT (min)	Delta RT (min)	Dwell Time (ms)	Q1 Pre Bias (V)	CE (V)	Q3 Pre Bias (V)
cadC+H	272	156	0.76	1.5	100.0	–30.0	–12.0	–27.0
cadC+Na	294	178	0.76	1.5	100.0	–19.0	–13.0	–11.0
dC+H	228	112	2.03	2.4	100.0	–27.0	–14.0	–18.0
dC+Na	250	134	2.03	2.4	100.0	–16.0	–13.0	–12.0
hmdC+H	258	142	2.35	2.4	100.0	–17.0	–11.0	–13.0
hmdC+Na	280	164	2.35	2.4	100.0	–19.0	–15.0	–16.0
mdC+H	242	126	4.70	1.2	100.0	–25.0	–13.0	–12.0
mdC+Na	264	148	4.70	1.2	100.0	–17.0	–14.0	–14.0
dG+H	268	152	5.10	1.2	100.0	–18.0	–11.0	–15.0
fdC+H	256	140	5.60	1.8	100.0	–26.0	–11.0	–12.0
fdC+Na	278	162	5.60	1.8	100.0	–18.0	–12.0	–10.0
dT+H	243	127	5.88	1.6	100.0	–19.0	–11.0	–12.0
dT+Na	265	149	5.88	1.6	100.0	–29.0	–12.0	–24.0
dA+H	252	136	6.37	1.6	100.0	–28.0	–15.0	–13.0

RT: retention time; CE: collision energy.

Table S5. Compound-dependent UHPLC-MS/MS parameters used for nucleosides quantification on gDNA. All the nucleosides were analyzed in the positive mode.

Compound	Precursor Ion (m/z)	Product Ion (m/z)	RT (min)	Delta RT (min)	CE (V)
cadC+H	272	156	1.5	1.0	12.0
cadC+Na	294	178	1.5	1.0	12.0
dC+H	228	112	3.8	1.0	10.0
dC+Na	250	134	3.8	1.0	10.0
hmdC+H	258	142	4.5	1.0	12.0
hmdC+Na	280	164	4.5	1.0	12.0
mdC+H	242	126	9.1	1.0	10.0
mdC+Na	264	148	9.1	1.0	10.0
dG+H	268	152	9.7	1.0	10.0
fdC+H	256	140	10.8	1.0	12.0
fdC+Na	278	162	10.8	1.0	12.0
dT+H	243	127	11.1	1.0	10.0
dT+Na	265	149	11.1	1.0	10.0
8-oxodG+H	284	168	12.2	1.0	10.0
dA+H	252	136	14.6	1.0	10.0

RT: retention time; CE: collision energy.

Supplementary Note 1. Preparation of model DNA.

1. 79 bp double-stranded 5hmC-containing DNA used for UHPLC-MS/MS analysis.

The 79 bp 5hmC-containing model DNA was annealed by 79 mer 5hmC-ODN and 79 mer ODN-R.

Name	Sequence (5' to 3')	Source
79 mer 5hmC-ODN	CCTCACCATCTCAACCAATATT**5hmC**G ATATTATGTCTACACGTTGGAGTTCCG TGTTATAATATTGAGGGAGAAGTGGT GA	Takara
79 mer ODN-R	TCACCACTTCTCCCTCAATATTATAAC ACGGAACTCCAACGTGTAGACATAAT ATCGAATATTGGTTGAGATGGTGAGG	Sangon Biotech

2. 13mer double-stranded 5fC-containing DNA used for UHPLC-MS/MS analysis.

The 13mer 5fC-containing model DNA used for UHPLC-MS/MS analysis was synthesized by primer extension method.

Name	Sequence (5' to 3')	Source
12 mer 5fC-F	GTCGACCGGATC	Sangon Biotech
17 mer 5fC-R	TTGGATCCGGTCGACTT	Sangon Biotech

Two DNA oligos (10 mM for each) were annealed in 1× annealing buffer containing 5 mM Tris-HCl (pH 7.5), 5 mM $MgCl_2$ and 50 mM NaCl and then incubated with 200 mM 5-formyl-2'-dCTP (Trilink Biotech) and 5 U Klenow Fragment 3'→5' exo– (New England Biolabs) in 50 ml of 1× NEBuffer 2 at 37 °C for 2 h. The product was purified with Micro Bio-Spin P-6 SSC column (Bio-Rad).
Final product sequence (5' to 3'):
GTCGACCGGATC**5fC**

3. 99 bp double-stranded 5fC-containing DNA used for UHPLC-MS/MS analysis.

The 99 bp 5fC-containing model DNA used for UHPLC-MS/MS analysis was synthesized by primer extension method.

Name	Sequence (5' to 3')	Source
99 mer 5fC template	CTCACTCACCTCCACCCTCTCACTACC TCACTCTTCCTCCTAACCCTCTCCAAC CACCTCTCCACCCTCCTAGATCTCTAC CTGACTGAGCGTGTGCGA	Sangon Biotech
5fC primer	TCGCACACGCTCAGTCAGGT	Sangon Biotech

1.5 mg 5fC template were added to a solution containing 10 mM primer, 150 mM 5-formyl-2'-dCTP (Trilink Biotech), dGTP, dATP, dTTP (New England Biolabs), 2.5 U DreamTaq polymerase (Thermo

Fisher) and 1× DreamTaq Buffer (Thermo Fisher). The solution was then incubated at 95 °C, 30 s; 68 °C, 60 s; 72 °C, 10 min and purified by Zymo-IC column with DNA Binding Buffer.

Final product sequence (5' to 3'):

TCGCACACGCTCAGTCAGGTAGAGAT**5fC**TAGGAGGGTGGAGAGGTGGTTGGAGAGGGTTAGG
AGGAAGAGTGAGGTAGTGAGAGGGTGGAGGTGAGTGAG

Supplementary Note 2. Preparation and sequences of 144mer synthetic 5hmC spike-in control.

Name	Sequence (5' to 3')	Source
74 mer 5hmC-F	AGAGAGCAAGCCGGCTATAGATGCTA CGTACAGTAGCAGCTGATCAAGACTG CTAAGGCCACAACCAGTTGGCG	IDT
90 mer 5hmC-R	ACTCTCACTCTCACCTCCATCTTACTT GTCTACCGAATCCTCACGTACTCACG ATTCGACCATCCACATTCGCCAACUG GUTGUGGCCTT	IDT

Two DNA oligos (10 mM for each) were annealed in 1× TruSeq annealing buffer containing 10 mM Tris-HCl (pH 7.9), 0.1 mM EDTA and 50 mM NaCl and then incubated with 400 mM 5-hydroxymethyl-2'-dCTP (Zymo Research), dGTP, dATP, dTTP (New England Biolabs) and 5 U Klenow Fragment 3'→5' exo– (New England Biolabs) in 50 ml of 1× NEBuffer 2 at 37 °C for 1 h. The product was purified by Zymo-IC column with Oligo Binding Buffer.
Final spike-in sequence (5' to 3'):
AGAGAGCAAGCCGGCTATAGATGCTACGTACAGTAGCAGCTGATCAAGACTGCTAAGGCCACAACCAGTTGGCGAATGTGGATGGT**5hmC**GAAT**5hmC**GTGAGTA**5hmC**GTGAGGATT**5hmC**GGTAGA**5hmC**AAGTAAGATGGAGGTGAGAGTGAGAGT

References:

(1) Liu, Y.; Siejka-Zielinska, P.; Velikova, G.; Bi, Y.; Yuan, F.; Tomkova, M.; Bai, C.; Chen, L.; Schuster-Bockler, B.; Song, C. X., Bisulfite-free direct detection of 5-methylcytosine and 5-hydroxymethylcytosine at base resolution. *Nat. Biotechnol.* **2019,** *37* (4), 424–429.

(2) Siejka-Zielinska, P.; Cheng, J. F.; Jackson, F.; Liu, Y. B.; Soonawalla, Z.; Reddy, S.; Silva, M.; Puta, L.; McCain, M. V.; Culver, E. L.; Bekkali, N.; Schuster-Bockler, B.; Palamara, P. F.; Mann, D.; Reeves, H.; Barnes, E.; Sivakumar, S.; Song, C. X., Cell-free DNA TAPS provides multimodal information for early cancer detection. *Sci. Adv.* **2021,** *7* (36), eabh0534.

(3) Zeng, H.; He, B.; Xia, B.; Bai, D.; Lu, X.; Cai, J.; Chen, L.; Zhou, A.; Zhu, C.; Meng, H.; Gao, Y.; Guo, H.; He, C.; Dai, Q.; Yi, C., Bisulfite-free, nanoscale analysis of 5-hydroxymethylcytosine at single base resolution. *J. Am. Chem. Soc.* **2018,** *140* (41), 13190–13194.

(4) Liu, Y.; Hu, Z.; Cheng, J.; Siejka-Zielinska, P.; Chen, J.; Inoue, M.; Ahmed, A. A.; Song, C. X., Subtraction-free and bisulfite-free specific sequencing of 5-methylcytosine and its oxidized derivatives at base resolution. *Nat. Commun.* **2021,** *12* (1), 618.

(5) Muller, C. A.; Boemo, M. A.; Spingardi, P.; Kessler, B. M.; Kriaucionis, S.; Simpson, J. T.; Nieduszynski, C. A., Capturing the dynamics of genome replication on individual ultra-long nanopore sequence reads. *Nat. Methods* **2019,** *16* (5), 429–436.

(6) Chen, S. F.; Zhou, Y. Q.; Chen, Y. R.; Gu, J., fastp: an ultra-fast all-in-one FASTQ preprocessor. *Bioinformatics* **2018,** *34* (17), 884–890.

(7) Li, H.; Durbin, R., Fast and accurate short read alignment with Burrows-Wheeler transform. *Bioinformatics* **2009,** *25* (14), 1754–1760.

(8) Dunham, I.; Kundaje, A.; Aldred, S. F.; Collins, P. J.; Davis, C.; Doyle, F.; Epstein, C. B.; Frietze, S.; Harrow, J.; Kaul, R.; Khatun, J.; Lajoie, B. R.; Landt, S. G.; Lee, B. K.; Pauli, F.; Rosenbloom, K. R.; Sabo, P.; Safi, A.; Sanyal, A.; Shoresh, N.; Simon, J. M.; Song, L.; Trinklein, N. D.; Altshuler, R. C.; Birney, E.; Brown, J. B.; Cheng, C.; Djebali, S.; Dong, X. J.; Dunham, I.; Ernst, J.; Furey, T. S.; Gerstein, M.; Giardine, B.; Greven, M.; Hardison, R. C.; Harris, R. S.; Herrero, J.; Hoffman, M. M.; Iyer, S.; Kellis, M.; Khatun, J.; Kheradpour, P.; Kundaje, A.; Lassmann, T.; Li, Q. H.; Lin, X.; Marinov, G. K.; Merkel, A.; Mortazavi, A.; Parker, S. C. J.; Reddy, T. E.; Rozowsky, J.; Schlesinger, F.; Thurman, R. E.; Wang, J.; Ward, L. D.; Whitfield, T. W.; Wilder, S. P.; Wu, W.; Xi, H. L. S.; Yip, K. Y.; Zhuang, J. L.; Bernstein, B. E.; Birney, E.; Dunham, I.; Green, E. D.; Gunter, C.; Snyder, M.; Pazin, M. J.; Lowdon, R. F.; Dillon, L. A. L.; Adams, L. B.; Kelly, C. J.; Zhang, J.; Wexler, J. R.; Green, E. D.; Good, P. J.; Feingold, E. A.; Bernstein, B. E.; Birney, E.; Crawford, G. E.; Dekker, J.; Elnitski, L.; Farnham, P. J.; Gerstein, M.; Giddings, M. C.; Gingeras, T. R.; Green, E. D.; Guigo, R.; Hardison, R. C.; Hubbard, T. J.; Kellis, M.; Kent, W. J.; Lieb, J. D.; Margulies, E. H.; Myers, R. M.; Snyder, M.; Stamatoyannopoulos, J. A.; Tenenbaum, S. A.; Weng, Z. P.; White, K. P.; Wold, B.; Khatun, J.; Yu, Y.; Wrobel, J.; Risk, B. A.; Gunawardena, H. P.; Kuiper, H. C.; Maier, C. W.; Xie, L.; Chen, X.; Giddings, M. C.; Bernstein, B. E.; Epstein, C. B.; Shoresh, N.; Ernst, J.; Kheradpour, P.; Mikkelsen, T. S.; Gillespie, S.; Goren, A.; Ram, O.; Zhang, X. L.; Wang, L.; Issner, R.; Coyne, M. J.; Durham, T.; Ku, M.; Truong, T.; Ward, L. D.; Altshuler, R. C.; Eaton, M. L.; Kellis, M.; Djebali, S.; Davis, C. A.; Merkel, A.; Dobin, A.; Lassmann, T.; Mortazavi, A.; Tanzer, A.; Lagarde, J.; Lin, W.; Schlesinger, F.; Xue, C. H.; Marinov, G. K.; Khatun, J.; Williams, B. A.; Zaleski, C.; Rozowsky, J.; Roeder, M.; Kokocinski, F.; Abdelhamid, R. F.; Alioto, T.; Antoshechkin, I.; Baer, M. T.; Batut, P.; Bell, I.; Bell, K.; Chakrabortty, S.; Chen, X.; Chrast, J.; Curado, J.; Derrien, T.; Drenkow, J.; Dumais, E.; Dumais, J.; Duttagupta, R.; Fastuca, M.; Fejes-Toth, K.; Ferreira, P.; Foissac, S.; Fullwood, M. J.; Gao, H.; Gonzalez, D.; Gordon, A.; Gunawardena, H. P.; Howald, C.; Jha, S.; Johnson, R.; Kapranov, P.; King, B.;

Kingswood, C.; Li, G. L.; Luo, O. J.; Park, E.; Preall, J. B.; Presaud, K.; Ribeca, P.; Risk, B. A.; Robyr, D.; Ruan, X. A.; Sammeth, M.; Sandhu, K. S.; Schaeffer, L.; See, L. H.; Shahab, A.; Skancke, J.; Suzuki, A. M.; Takahashi, H.; Tilgner, H.; Trout, D.; Walters, N.; Wang, H. E.; Wrobel, J.; Yu, Y. B.; Hayashizaki, Y.; Harrow, J.; Gerstein, M.; Hubbard, T. J.; Reymond, A.; Antonarakis, S. E.; Hannon, G. J.; Giddings, M. C.; Ruan, Y. J.; Wold, B.; Carninci, P.; Guigo, R.; Gingeras, T. R.; Rosenbloom, K. R.; Sloan, C. A.; Learned, K.; Malladi, V. S.; Wong, M. C.; Barber, G.; Cline, M. S.; Dreszer, T. R.; Heitner, S. G.; Karolchik, D.; Kent, W. J.; Kirkup, V. M.; Meyer, L. R.; Long, J. C.; Maddren, M.; Raney, B. J.; Furey, T. S.; Song, L. Y.; Grasfeder, L. L.; Giresi, P. G.; Lee, B. K.; Battenhouse, A.; Sheffield, N. C.; Simon, J. M.; Showers, K. A.; Safi, A.; London, D.; Bhinge, A. A.; Shestak, C.; Schaner, M. R.; Kim, S. K.; Zhang, Z. Z. Z.; Mieczkowski, P. A.; Mieczkowska, J. O.; Liu, Z.; McDaniell, R. M.; Ni, Y. Y.; Rashid, N. U.; Kim, M. J.; Adar, S.; Zhang, Z. C.; Wang, T. Y.; Winter, D.; Keefe, D.; Birney, E.; Iyer, V. R.; Lieb, J. D.; Crawford, G. E.; Li, G. L.; Sandhu, K. S.; Zheng, M. Z.; Wang, P.; Luo, O. J.; Shahab, A.; Fullwood, M. J.; Ruan, X. A.; Ruan, Y. J.; Myers, R. M.; Pauli, F.; Williams, B. A.; Gertz, J.; Marinov, G. K.; Reddy, T. E.; Vielmetter, J.; Partridge, E. C.; Trout, D.; Varley, K. E.; Gasper, C.; Bansal, A.; Pepke, S.; Jain, P.; Amrhein, H.; Bowling, K. M.; Anaya, M.; Cross, M. K.; King, B.; Muratet, M. A.; Antoshechkin, I.; Newberry, K. M.; McCue, K.; Nesmith, A. S.; Fisher-Aylor, K. I.; Pusey, B.; DeSalvo, G.; Parker, S. L.; Balasubramanian, S.; Davis, N. S.; Meadows, S. K.; Eggleston, T.; Gunter, C.; Newberry, J. S.; Levy, S. E.; Absher, D. M.; Mortazavi, A.; Wong, W. H.; Wold, B.; Blow, M. J.; Visel, A.; Pennachio, L. A.; Elnitski, L.; Margulies, E. H.; Parker, S. C. J.; Petrykowska, H. M.; Abyzov, A.; Aken, B.; Barrell, D.; Barson, G.; Berry, A.; Bignell, A.; Boychenko, V.; Bussotti, G.; Chrast, J.; Davidson, C.; Derrien, T.; Despacio-Reyes, G.; Diekhans, M.; Ezkurdia, I.; Frankish, A.; Gilbert, J.; Gonzalez, J. M.; Griffiths, E.; Harte, R.; Hendrix, D. A.; Howald, C.; Hunt, T.; Jungreis, I.; Kay, M.; Khurana, E.; Kokocinski, F.; Leng, J.; Lin, M. F.; Loveland, J.; Lu, Z.; Manthravadi, D.; Mariotti, M.; Mudge, J.; Mukherjee, G.; Notredame, C.; Pei, B. K.; Rodriguez, J. M.; Saunders, G.; Sboner, A.; Searle, S.; Sisu, C.; Snow, C.; Steward, C.; Tanzer, A.; Tapanari, E.; Tress, M. L.; van Baren, M. J.; Walters, N.; Washietl, S.; Wilming, L.; Zadissa, A.; Zhang, Z. D.; Brent, M.; Haussler, D.; Kellis, M.; Valencia, A.; Gerstein, M.; Reymond, A.; Guigo, R.; Harrow, J.; Hubbard, T. J.; Landt, S. G.; Frietze, S.; Abyzov, A.; Addleman, N.; Alexander, R. P.; Auerbach, R. K.; Balasubramanian, S.; Bettinger, K.; Bhardwaj, N.; Boyle, A. P.; Cao, A. R.; Cayting, P.; Charos, A.; Cheng, Y.; Cheng, C.; Eastman, C.; Euskirchen, G.; Fleming, J. D.; Grubert, F.; Habegger, L.; Hariharan, M.; Harmanci, A.; Iyengar, S.; Jin, V. X.; Karczewski, K. J.; Kasowski, M.; Lacroute, P.; Lam, H.; Lamarre-Vincent, N.; Leng, J.; Lian, J.; Lindahl-Allen, M.; Min, R. Q.; Miotto, B.; Monahan, H.; Moqtaderi, Z.; Mu, X. M. J.; O'Geen, H.; Ouyang, Z. Q.; Patacsil, D.; Pei, B. K.; Raha, D.; Ramirez, L.; Reed, B.; Rozowsky, J.; Sboner, A.; Shi, M. Y.; Sisu, C.; Slifer, T.; Witt, H.; Wu, L. F.; Xu, X. Q.; Yan, K. K.; Yang, X. Q.; Yip, K. Y.; Zhang, Z. D.; Struhl, K.; Weissman, S. M.; Gerstein, M.; Farnham, P. J.; Snyder, M.; Tenenbaum, S. A.; Penalva, L. O.; Doyle, F.; Karmakar, S.; Landt, S. G.; Bhanvadia, R. R.; Choudhury, A.; Domanus, M.; Ma, L. J.; Moran, J.; Patacsil, D.; Slifer, T.; Victorsen, A.; Yang, X. Q.; Snyder, M.; White, K. P.; Auer, T.; Centanin, L.; Eichenlaub, M.; Gruhl, F.; Heermann, S.; Hoeckendorf, B.; Inoue, D.; Kellner, T.; Kirchmaier, S.; Mueller, C.; Reinhardt, R.; Schertel, L.; Schneider, S.; Sinn, R.; Wittbrodt, B.; Wittbrodt, J.; Weng, Z. P.; Whitfield, T. W.; Wang, J.; Collins, P. J.; Aldred, S. F.; Trinklein, N. D.; Partridge, E. C.; Myers, R. M.; Dekker, J.; Jain, G.; Lajoie, B. R.; Sanyal, A.; Balasundaram, G.; Bates, D. L.; Byron, R.; Canfield, T. K.; Diegel, M. J.; Dunn, D.; Ebersol, A. K.; Frum, T.; Garg, K.; Gist, E.; Hansen, R. S.; Boatman, L.; Haugen, E.; Humbert, R.; Jain, G.; Johnson, A. K.; Johnson, E. M.; Kutyavin, T. V.; Lajoie, B. R.; Lee, K.; Lotakis, D.; Maurano, M. T.; Neph, S. J.; Neri, F. V.; Nguyen, E. D.; Qu, H. Z.; Reynolds, A. P.; Roach, V.; Rynes, E.; Sabo, P.; Sanchez, M. E.; Sandstrom, R. S.; Sanyal, A.; Shafer, A. O.; Stergachis, A. B.; Thomas, S.; Thurman, R. E.; Vernot, B.; Vierstra, J.; Vong, S.; Wang, H.; Weaver, M. A.; Yan, Y. Q.; Zhang, M. H.; Akey, J. M.; Bender, M.; Dorschner, M. O.; Groudine, M.; MacCoss, M. J.; Navas, P.; Stamatoyannopoulos, G.; Kaul, R.; Dekker, J.;

Stamatoyannopoulos, J. A.; Dunham, I.; Beal, K.; Brazma, A.; Flicek, P.; Herrero, J.; Johnson, N.; Keefe, D.; Lukk, M.; Luscombe, N. M.; Sobral, D.; Vaquerizas, J. M.; Wilder, S. P.; Batzoglou, S.; Sidow, A.; Hussami, N.; Kyriazopoulou-Panagiotopoulou, S.; Libbrecht, M. W.; Schaub, M. A.; Kundaje, A.; Hardison, R. C.; Miller, W.; Giardine, B.; Harris, R. S.; Wu, W.; Bickel, P. J.; Banfai, B.; Boley, N. P.; Brown, J. B.; Huang, H. Y.; Li, Q. H.; Li, J. J.; Noble, W. S.; Bilmes, J. A.; Buske, O. J.; Hoffman, M. M.; Sahu, A. D.; Kharchenko, P. V.; Park, P. J.; Baker, D.; Taylor, J.; Weng, Z. P.; Iyer, S.; Dong, X. J.; Greven, M.; Lin, X. Y.; Wang, J.; Xi, H. L. S.; Zhuang, J. L.; Gerstein, M.; Alexander, R. P.; Balasubramanian, S.; Cheng, C.; Harmanci, A.; Lochovsky, L.; Min, R.; Mu, X. M. J.; Rozowsky, J.; Yan, K. K.; Yip, K. Y.; Birney, E.; Consortium, E. P., An integrated encyclopedia of DNA elements in the human genome. *Nature* **2012,** *489* (7414), 57–74.

(9) Amemiya, H. M.; Kundaje, A.; Boyle, A. P., The ENCODE blacklist: identification of Problematic Regions of the Genome. *Sci. Rep.* **2019,** *9*, 9354.

(10) Incarnato, D.; Krepelova, A.; Neri, F., High-throughput single nucleotide variant discovery in E14 mouse embryonic stem cells provides a new reference genome assembly. *Genomics* **2014,** *104* (2), 121–127.

(11) Rosenbloom, K. R.; Armstrong, J.; Barber, G. P.; Casper, J.; Clawson, H.; Diekhans, M.; Dreszer, T. R.; Fujita, P. A.; Guruvadoo, L.; Haeussler, M.; Harte, R. A.; Heitner, S.; Hickey, G.; Hinrichs, A. S.; Hubley, R.; Karolchik, D.; Learned, K.; Lee, B. T.; Li, C. H.; Miga, K. H.; Nguyen, N.; Paten, B.; Raney, B. J.; Smit, A. F. A.; Speir, M. L.; Zweig, A. S.; Haussler, D.; Kuhn, R. M.; Kent, W. J., The UCSC Genome Browser database: 2015 update. *Nucleic Acids Res.* **2015,** *43* (D1), D670–D681.

(12) Yu, M.; Hon, G. C.; Szulwach, K. E.; Song, C. X.; Zhang, L.; Kim, A.; Li, X.; Dai, Q.; Shen, Y.; Park, B.; Min, J. H.; Jin, P.; Ren, B.; He, C., Base-resolution analysis of 5-hydroxymethylcytosine in the mammalian genome. *Cell* **2012,** *149* (6), 1368–1380.

(13) Schutsky, E. K.; DeNizio, J. E.; Hu, P.; Liu, M. Y.; Nabel, C. S.; Fabyanic, E. B.; Hwang, Y.; Bushman, F. D.; Wu, H.; Kohli, R. M., Nondestructive, base-resolution sequencing of 5-hydroxymethylcytosine using a DNA deaminase. *Nat. Biotechnol.* **2018,** *36* (11), 1083–1090.

(14) Wen, L.; Li, X. L.; Yan, L. Y.; Tan, Y. X.; Li, R.; Zhao, Y. Y.; Wang, Y.; Xie, J. C.; Zhang, Y.; Song, C. X.; Yu, M.; Liu, X. M.; Zhu, P.; Li, X. Y.; Hou, Y.; Guo, H. S.; Wu, X. L.; He, C.; Li, R. Q.; Tang, F. C.; Qiao, J., Whole-genome analysis of 5-hydroxymethylcytosine and 5-methylcytosine at base resolution in the human brain. *Genome Biol.* **2014,** *15* (3), R49.

(15) Zhao, H.; Sun, Z. F.; Wang, J.; Huang, H. J.; Kocher, J. P.; Wang, L. G., CrossMap: a versatile tool for coordinate conversion between genome assemblies. *Bioinformatics* **2014,** *30* (7), 1006–1007.

(16) Bogu, G. K.; Vizan, P.; Stanton, L. W.; Beato, M.; Di Croce, L.; Marti-Renom, M. A., Chromatin and RNA maps reveal regulatory long noncoding RNAs in mouse. *Mol. Cell. Biol.* **2016,** *36* (5), 809–819.

(17) Quinlan, A. R.; Hall, I. M., BEDTools: a flexible suite of utilities for comparing genomic features. *Bioinformatics* **2010,** *26* (6), 841–842.

(18) Ramirez, F.; Ryan, D. P.; Gruning, B.; Bhardwaj, V.; Kilpert, F.; Richter, A. S.; Heyne, S.; Dundar, F.; Manke, T., deepTools2: a next generation web server for deep-sequencing data analysis. *Nucleic Acids Res.* **2016,** *44* (W1), W160–W165.

(19) Kent, W. J.; Zweig, A. S.; Barber, G.; Hinrichs, A. S.; Karolchik, D., BigWig and BigBed: enabling browsing of large distributed datasets. *Bioinformatics* **2010,** *26* (17), 2204–2207.

(20) Robinson, J. T.; Thorvaldsdottir, H.; Winckler, W.; Guttman, M.; Lander, E. S.; Getz, G.; Mesirov, J. P., Integrative genomics viewer. *Nat. Biotechnol.* **2011,** *29* (1), 24–26.

(21) Song, C. X.; Szulwach, K. E.; Dai, Q.; Fu, Y.; Mao, S. Q.; Lin, L.; Street, C.; Li, Y.; Poidevin, M.; Wu, H.; Gao, J.; Liu, P.; Li, L.; Xu, G. L.; Jin, P.; He, C., Genome-wide profiling of 5-formylcytosine reveals its roles in epigenetic priming. *Cell* **2013,** *153* (3), 678–691.

(22) Liu, P. Y.; Jiang, N.; Zhang, J.; Wei, X.; Lin, H. H.; Yu, X. Q., The oxidative damage of plasmid DNA by ascorbic acid derivatives *in vitro*: the first research on the relationship between the structure of ascorbic acid and the oxidative damage of plasmid DNA. *Chem. Biodivers.* **2006,** *3* (9), 958–966.

(23) Abel, G. R., Jr.; Calabrese, Z. A.; Ayco, J.; Hein, J. E.; Ye, T., Measuring and suppressing the oxidative damage to DNA during Cu(I)-catalyzed azide-alkyne cycloaddition. *Bioconjug. Chem.* **2016,** *27* (3), 698–704.

(24) Qiu, J. C.; Pradhan, P. P.; Blanck, N. B.; Bobbitt, J. M.; Bailey, W. F., Selective oxoammonium salt oxidations of alcohols to aldehydes and aldehydes to carboxylic acids. *Org. Lett.* **2012,** *14* (1), 350–353.

(25) Mercadante, M. A.; Kelly, C. B.; Bobbitt, J. M.; Tilley, L. J.; Leadbeater, N. E., Synthesis of 4-acetamido-2,2,6,6-tetramethylpiperidine-1-oxoammonium tetrafluoroborate and 4-acetamido-(2,2,6,6-tetramethyl-piperidin-1-yl)oxyl and their use in oxidative reactions. *Nat. Protoc.* **2013,** *8* (4), 666–676.

(26) Hussein, A. A.; Al-Hadedi, A. A. M.; Mahrath, A. J.; Moustafa, G. A. I.; Almalki, F. A.; Alqahtani, A.; Shityakov, S.; Algazally, M. E., Mechanistic investigations on Pinnick oxidation: a density functional theory study. *Roy. Soc. Open Sci.* **2020,** *7* (2), 191568.

(27) Dalcanale, E.; Montanari, F., Selective oxidation of aldehydes to carboxylic acids with sodium chlorite-hydrogen peroxide. *J. Org. Chem.* **1986,** *51* (4), 567–569.

(28) Fang, X. Q.; Bandarage, U. K.; Wang, T.; Schroeder, J. D.; Garvey, D. S., First examples of oxidizing aldehydes to carboxylic acids in the presence of a tertiary disulfide functional group: synthesis of novel diacid-disulfides. *Synlett* **2003,** *4* (4), 489–492.
